# Inhibition of autocrine HGF maturation overcomes cetuximab resistance in colorectal cancer

**DOI:** 10.1007/s00018-023-05071-5

**Published:** 2024-01-12

**Authors:** Vivian Truong Jones, Ramona Graves-Deal, Zheng Cao, Galina Bogatcheva, Marisol A. Ramirez, Sarah J. Harmych, James N. Higginbotham, Vineeta Sharma, Vishnu C. Damalanka, Claudia C. Wahoski, Neeraj Joshi, Maria Johnson Irudayam, Joseph T. Roland, Gregory D. Ayers, Qi Liu, Robert J. Coffey, James W. Janetka, Bhuminder Singh

**Affiliations:** 1 Department of Medicine, Vanderbilt University Medical Center, 10465J, MRB IV, 2213 Garland Avenue, Nashville, TN 37232-0441 USA; 2 Department of Pharmacology, Vanderbilt University, Nashville, TN 37232 USA; 3 Department of Biostatistics, Vanderbilt University Medical Center, Nashville, TN 37232 USA; 4 Center for Quantitative Sciences, Vanderbilt University Medical Center, Nashville, TN 37232 USA; 5 Department of Cell and Developmental Biology, Vanderbilt University, Nashville, TN 37232 USA; 6 Department of Biochemistry and Molecular Biophysics, Washington University School of Medicine, Saint Louis, MO 63110 USA; 7 Program in Cancer Biology, Vanderbilt University, Nashville, TN 37232 USA; 8 Department of Surgery, Vanderbilt University Medical Center, Nashville, TN 37232 USA; 9 Epithelial Biology Center, Vanderbilt University Medical Center, Nashville, TN 37232 USA

**Keywords:** Colorectal cancer, Drug resistance, Cetuximab, Crizotinib, EGFR, MET, HGF, HAI-1, 3D culture, Protease inhibition

## Abstract

Although amplifications and mutations in receptor tyrosine kinases (RTKs) act as bona fide oncogenes, in most cancers, RTKs maintain moderate expression and remain wild-type. Consequently, cognate ligands control many facets of tumorigenesis, including resistance to anti-RTK therapies. Herein, we show that the ligands for the RTKs MET and RON, HGF and HGFL, respectively, are synthesized as inactive precursors that are activated by cellular proteases. Our newly generated HGF/HGFL protease inhibitors could overcome both de novo and acquired cetuximab resistance in colorectal cancer (CRC). Conversely, HGF overexpression was necessary and sufficient to induce cetuximab resistance and loss of polarity. Moreover, HGF-induced cetuximab resistance could be overcome by the downstream MET inhibitor, crizotinib, and upstream protease inhibitors. Additionally, HAI-1, an endogenous inhibitor of HGF proteases, (i) was downregulated in CRC, (ii) exhibited increased genomic methylation that correlated with poor prognosis, (iii) HAI-1 expression correlated with cetuximab response in a panel of cancer cell lines, and (iv) exogenous addition of recombinant HAI-1 overcame cetuximab resistance in CC-HGF cells. Thus, we describe a targetable, autocrine HAI-1/Protease/HGF/MET axis in cetuximab resistance in CRC.

**Supplementary Information:**

The online version contains supplementary material available at 10.1007/s00018-023-05071-5.

## Background

Colorectal cancer (CRC) is the second deadliest cancer both in the United States and globally [1]. About 20% of patients are diagnosed with metastatic disease despite improvements in screening [2]. Often, these individuals will experience no signs or symptoms of the disease until it is discovered at a late stage. Although early stages of CRC have promising 5-year survival rates, the 5-year survival rate for stage IV is a meager 14% [2]. The addition of targeted therapies such as cetuximab and panitumumab has prolonged overall survival rates for metastatic CRC to a median of 30 months compared to 20 months with conventional chemotherapy [3–7].

Cetuximab and panitumumab are monoclonal antibodies used to inhibit epidermal growth factor receptor (EGFR) in RAS wild-type metastatic CRC [7]. Dysregulation of EGFR signaling plays a crucial role in carcinogenesis. EGFR activation turns on the mitogen-activated protein kinase (MAPK) signaling pathway to promote proliferation, inhibit apoptosis, and induce invasion and metastasis [3]. Acquired therapeutic resistance is a complication that all patients will inevitably encounter and limits the success of these treatments [8]. Resistance may arise from mechanisms that compensate for diminished EGFR signaling [8, 9]. Genetic modes of resistance such as mutations in EGFR and downstream effectors (e.g., RAS/RAF) are common and have been included in the recommendation to exclude individuals carrying certain genetic lesions. EGFR/RAS/RAF mutations render anti-EGFR therapies ineffective and instead add to the adverse effects of the treatment experienced by the individuals undergoing treatment. Non-genetic modes of anti-EGFR resistance remain understudied and poorly defined [9].

We have previously uncovered a new non-genetic mode of cetuximab resistance due to the increased phosphorylation of the receptor tyrosine kinases (RTKs) MET and RON in our 3D cultures of CRC cell lines [10]. Moreover, we were able to overcome cetuximab resistance by inhibition of MET/RON with crizotinib treatment in our in vitro cultures and in our in vivo xenograft studies [11]. MET activation drives the malignant progression of CRC by promoting signaling cascades that mainly affect cancer cells’ survival, proliferation, migration, and invasion. Crosstalk between the EGFR axis and the MET/HGF axis—as defined by the proximal events to the respective RTKs—forms the basis for resistance against anti-EGFR therapy as MET activation also activates the MAPK signaling pathway.

Because no MET/RON amplifications or activating mutations were identified in our cetuximab-resistance studies, we decided to focus on their ligands and their regulation. In this report, we aimed to determine whether upstream activation and maturation of the MET ligand, hepatocyte growth factor (HGF), is an important mechanism of anti-EGFR drug resistance. We generated an HGF overexpressing cell line that resulted in its cetuximab resistance (CC-HGF). We demonstrate that CC-HGF and our other cetuximab-resistant lines have a reduced colony count when treated with cetuximab and crizotinib together. Further, inhibitors of HGF processing and maturation (ZFH7116, VD2173, MM3122, VD5064) also reduced colony count in these same cell lines. We also identified HAI-1 as an endogenous negative regulator that predicts cetuximab response. Ultimately, ligand processing and maturation may be an important targetable pathway in cetuximab resistance.

## Methods

### Reagents

PureCol bovine type I collagen was purchased from Advanced Biomatrix, Inc. (San Diego, CA, USA, #5005–100 ml). Unless specified otherwise, all cell culture components were purchased from Hyclone Laboratories, Inc. (Omaha, NE, USA). DMEM and 10X DMEM were purchased from Gibco (#11,430–030). Collagenase, type I was purchased from MilliporeSigma (Temecula, CA, USA, #234,153). Recombinant human HGF was purchased from R&D Systems (Minneapolis, MN, USA, #294-HG/CF). Recombinant human HAI-1 was purchased from SinoBiological (Houston, TX, USA, #11,742-H08H). Propidium iodide was purchased from Invitrogen (Carlsbad, CA, USA, #P3566). Sodium Hydroxide 10 Normal was purchased from VWR Chemicals BDH (Radnor, PA, USA, #BDH3247-1). Crizotinib was purchased from MilliporeSigma (Temecula, CA, USA, #PZ0191). Cetuximab was purchased from Eli Lilly and Company. ZFH7116, VD2173, MM3122, VD4162, and VD5064 were generated in the lab of James Janetka at Washington University in St. Louis, MO.

### Cell lines, lentiviral assembly, and lentiviral vector transduction

The HCA-7 cell line was obtained from Susan Kirkland (Imperial Cancer Research Fund); its derivatives (SC, CC, and CC-CR) have been described earlier [10, 12]. CC were stably transduced with an HGF lentiviral vector for overexpression. The human HGF-expressing lentiviral vector, encoded for human HGF and GFP-Spark Tag, was purchased from SinoBiological (HG10463-ACGLN). Twenty-four hours before transfection, 2.5 × 10^6^ Phoenix cells were plated on a 10 cm dish for lentiviral assembly. HGF-containing or control lentiviral vectors were co-transfected with packing plasmids into Phoenix cells to produce lentivirus using Genecopoeia Lenti-Pac HIV packaging kit (cat# HPK-LvTR-20) according to the manufacturer’s instructions. Approximately 2.5 × 10^6^ CC cells were plated on 10 cm dish. Media containing viral particles from the Phoenix cells was filtered with Steriflip (Millipore, Billerica, MA). The recipient CC cells were infected three times with media containing viral particles, and pools were selected upon the addition of 1 μg/ml of puromycin. The transfected cells were observed under a fluorescence microscope (Zeiss) 7–10 days after transfection. HGF-expressing pools were then FACS cloned in 96-well dishes and expanded to stable clones.

### Cell culture (maintenance of cells on plastic)

Unless otherwise indicated, all cell lines were maintained in DMEM supplemented with 10% bovine growth serum, non-essential amino acids, L-glutamine, and penicillin/streptomycin. Additionally, the CC-CR culture medium contained cetuximab (3 μg/ml) which was removed during our 3D experiments.

### 3D type I collagen culture

The concentration of collagen was 2 mg/ml (PureCol^®^ Type I Collagen #5005) in 1X DMEM (diluted from 10X DMEM with sterile water) and supplemented with 10% fetal bovine serum and pH adjusted to neutral with 10 N NaOH. For a 12-well dish, three distinct collagen layers at 400 µl each were layered on top of each other (collagen layer, single-cell collagen suspension layer, and collagen layer). Cells were seeded at 5000 cells/ml (2000 cells/well) for colony counting experiments and at 250,000 cells/ml (100,000 cells/well) for western blot and FACS. After the last collagen layer was polymerized, each well was supplemented with 400 µl of medium with or without treatment. Medium changes occurred every two to three days. Colonies were observed and counted after a minimum of 14 days.

Colony Count Assay: Colonies were counted using GelCount (Oxford Optronix) with identical acquisition and analysis settings. SC, CC, and CC-HGF colonies were typically counted at ~ 14 days, while CC-CR colonies were counted at ~ 20 days.

### Immunoblotting from 3D cultures

After a week of growth, the cells were treated for the indicated periods of time. The middle cell-containing collagen layer from the 3D collagen cultures was separated from outer collagen layers and placed into 600 µl of fresh lysis buffer and incubated for two hours at 4 °C while rotating. Lysates were cleared by centrifugation at 14,000 rpm for 15 min. Supernatants were diluted with 4X Laemmli sample buffer containing 5% β-mercaptoethanol, boiled for 5 min at 95 °C, separated on a 7.5% SDS/PAGE, and electro-blotted onto nitrocellulose membranes. Membranes were blocked with 5% milk in TBS containing 0.1% Tween 20 (TBS-T) or in Odyssey blocking buffer for 2–4 h at room temperature while rocking. Primary and secondary antibodies were incubated for one hour at room temperature while rocking followed by 4 × 15 min TBS-T washes. Blots were developed using LI-COR Odyssey or enhanced ECL.

### Cell lysis, immunoblotting, and immunoprecipitation from 2D cultures

Cells were seeded at 200,000 cells per 10 cm dish. On day four, the media was changed, and cells were treated with 50 µM of each indicated inhibitor. Conditioned media was collected and filtered 48 h later. GFP-Trap™ Magnetic Particles M-270 (Proteintech/ChromoTek #gtdk-20) were added to the conditioned media and incubated at 4 °C overnight while rocking then washed three times the following day. Beads were resuspended in 2X Laemmli sample buffer containing 5% β-mercaptoethanol. Cells were lysed in 1 ml of lysis buffer and processed for immunoblotting as described for 3D immunoblotting above.

### Lysis buffer composition

50 mM HEPES (pH 7.5), 150 mM NaCl, 1% Triton X-100, 1 mM EDTA, 10% glycerol, and 10 mM sodium pyrophosphate. Following reagents were added just before lysis at the indicated concentrations: 2 mM sodium orthovanadate, 10 mM sodium fluoride, 1 mM PMSF, 5 μg/ml leupeptin, 5 μg/ml pepstatin and 5 μg/ml aprotinin.

### Antibodies

Anti-MET (R&D #AF276 goat antibody, 1:2,000); anti-ERK1/2 (Cell Signaling #9102 rabbit antibody, 1:1,000); anti-AKT (Cell Signaling #9272 rabbit antibody, 1:1,000); anti-human HGF (R&D # AF-294-NA goat antibody 0.5 µg/ml); anti-pMET-Y1234/1235 (Cell Signaling #3077 rabbit antibody, 1:1,000); anti-pERK1/2 (Cell Signaling #9101 rabbit antibody, 1:1,000); anti-pAKT-serine473 (Cell Signaling #9271 rabbit antibody, 1:1,000); anti-tubulin (Calbiochem #CP06 mouse antibody, 1:5000). Goat anti-mouse (IRDye 680LT IgG H + L, LI-COR P/N 926–68,020, 1:15,000); donkey anti-goat (IRDye 680LT IgG H + L, LI-COR P/N 926–68,024, 1:15,000); donkey anti-rabbit (IRDye 800CW IgG H + L, LI-COR P/N 926–32,213, 1:15,000); mouse TrueBlot ULTRA (anti-mouse IgG HRP, Rockland Inc. #18–8817-33, 1:2,000); rabbit TrueBlot (anti-rabbit IgG HRP, Rockland Inc. #18–8816-33, 1:2,000); goat TrueBlot (anti-goat IgG HRP, Rockland Inc. #18–8814-33, 1:2,000).

### 2D/3D CellTiterGlo assay

To assess cell viability and determine IC50 values, Promega’s CellTiter-Glo ATP-based assay was utilized. Cells were seeded at 10,000 cells/well in a 96-well plate (Falcon 96-well Clear Flat Bottom TC-treated Culture Microplate). Cetuximab was added on the day of seeding and the plate was incubated for 72 h. Plates were removed from the incubator and 50 µl of CellTiter-Glo reagent was added directly into the wells. Plates were incubated on a shaker at room temperature for 30 min. Luminescence was measured on a Synergy H1 reader (Biotek). Luminescence readings were normalized as a percentage of the control. Dose–response curves and IC50 values were estimated using the R package drc v3.0–1 [13].

### Immunofluorescence

After 14–20 days of culture, middle layers of mature collagen cultures was teased out with tweezers and washed with PBS three times, five min each. Collagen layer was then fixed with 4% PFA at 4 °C for 1 h with gentle rocking. Samples were washed again with PBS three times, 5 min each and then placed in IF buffer overnight at 4 °C with gentle rocking (IF buffer: 1% BSA, 0.1% Triton X-100, 0.01% Sodium azide in PBS). All remaining steps were performed in IF buffer at room temperature with gentle rocking. Samples were then blocked with 3% normal donkey serum for 2–4 h. Primary antibodies were added for 1 h followed by three 15 min washes. Secondary antibodies were added for 1 h followed by three 15 min washes. DAPI and Phalloidin dyes were added at secondary antibody incubation stage. Stained samples were then placed in chamber slides with coverslip bottoms when ready to image.

### FACS analysis

After 1 week of growth, cells were incubated with indicated drugs for 24 h. The middle cell-containing collagen layer from the 3D collagen cultures was removed and incubated with 1 ml of type I collagenase (3 mg/ml in DMEM) for 30 min at 37 °C with gentle rocking. After this incubation, 300 μl of 10X TrypLE and 15 μl of 0.5 mM EDTA were added and samples incubated under the same conditions for another 30 min. Cells were harvested by spinning at 200 *g* for 10 min at 4 °C then resuspended in 400 μl PBS by passing through a 25-gauge needle 5–7 times. Cells were fixed by adding 5 ml of 70% ethanol (kept at –20 °C) dropwise while vortexing. It was stored overnight at 4 °C. Fixed cells were re-suspended in ~ 7 ml PBS and mixed by inverting 1–2 times then equilibrated for 5 min by gentle rocking. Cells were spun down at 1000 *g* for 10 min at 4 °C then re-suspended in 500 μl PBS (containing 25 μl of 1 mg/ml propidium iodide and 10 μl of 10 mg/ml RNase A). They were incubated for 1 h in Eppendorf tubes rocking at 37 °C. Samples were stored overnight at 4 °C and then the cell cycle was analyzed by flow cytometry. Samples were analyzed using BD™ LSRII (BD Biosciences, San Jose, CA) and analyzed with BD FACSDiva software (ver. 8.0.3).

### Colony circularity analysis

Cells were seeded and treated on a 12-well plate at 10,000 cells/ml in 3D in type I collagen. Colonies were grown under treatment for 7 days and images were captured on day 7 using the MuviCyte Live-Cell Imaging System (PerkinElmer). To prevent bias, nine images were captured per well with a 4 × objective lens. Images were analyzed using a combination of machine learning [14] and image analysis (MATLAB version: 9.13.0 (R2021a), Natick, Massachusetts: The MathWorks Inc.; 2022.). Machine learning was applied on all collected images to identify only those in-focus colonies based on image texture (Ilastik v1.4.0). Resulting probability maps were the basis of image masks demarking only in-focus colonies. Characteristics of the identified colonies were recorded to quantify colony circularity with the equation: (4*Area*π)/(Perimeter^2^) and colony roundness with the equation: (4*Area)/(π)*(Major Axis length^2^). Density plot and data analysis were conducted in R software (https://www.r-project.org/), and figures were produced using the “ggplot2” R package [15].

### TCGA data

The publicly available TCGA database was used to investigate gene expression levels in colorectal cancer and normal tissue. Wilcoxon rank sum test was used to compare expression differences. To stratify the colorectal tumor samples, the Consensus Molecular Subtypes (CMS) classification from the Colorectal Cancer Subtyping Consortium (CRCSC) was applied using the “CMSCaller” R package [16]. Subtypes are as follows: CMS1-MSI/immune, CMS2-canonical, CMS3-epithelial/metabolic, CMS4-mesenchymal, and mixed. TCGA-COAD database was also used to analyze DNA methylation and survival for SPINT1/HAI-1. Survival analysis was performed using the Shiny Methylation Analysis Resource Tool (SMART) [17]. Log-rank test was used to analyze the survival data.

### Statistical analysis

Each experiment was performed at least three times in triplicates. Graphs in this study were plotted using the “ggplot2” R package [15]. The statistical tools and methods for each analysis are described in the figure legends.

## Results

### HGF/HGFL protease inhibitors overcome de novo and acquired modes of cetuximab resistance

**Fig. 1:**
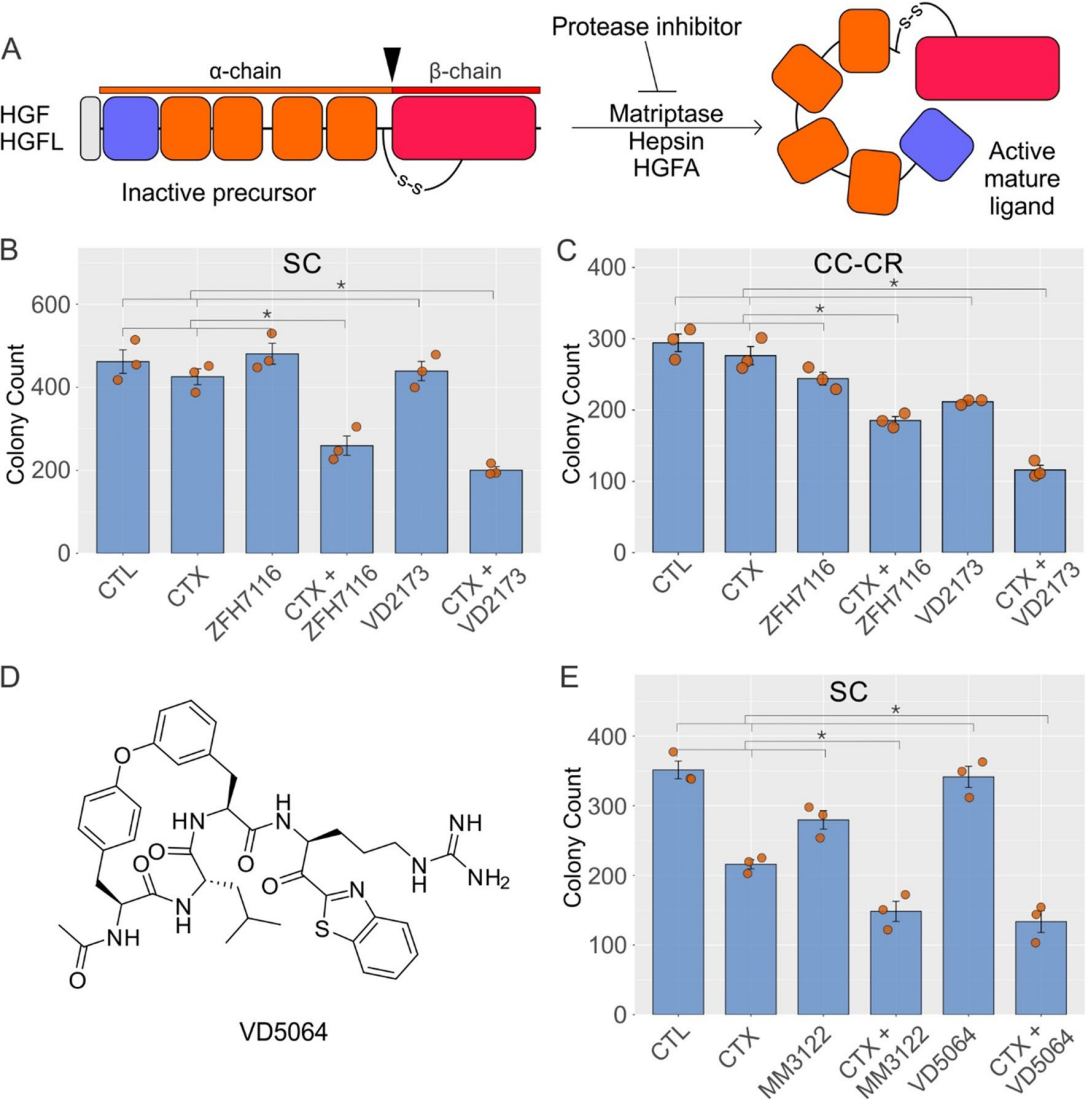
HGF/HGFL protease inhibitors overcome de novo and acquired modes of cetuximab resistance. **A** Domain organization and maturation of human HGF/HGFL. The 728/711-amino acid (aa) pre-propeptide consists of a 31/18-aa signal peptide (grey), a 87/85-aa PAN domain (purple), four 87–89-aa long Kringle domains (orange), and a 227/226-aa serine protease homology (SPH) domain (red). Ligand maturation requires proteolytic cleavage between α and β chains, as indicated by the arrowhead; inhibitors of these proteases (Matriptase, Hepsin, and HGFA) consequently limit availability of biologically active HGF/HGFL. Two thousand **B** HCA-7-derived spiky clone (SC) and **C** cetuximab-resistant cystic clone (CC-CR) were seeded in 3D in type I collagen and incubated with cetuximab (CTX, 3 μg/ml) alone or in combination with HGF/HGFL protease inhibitors ZFH7116 (50 μM) and VD2173 (50 μM) for 14–21 days. Colony counts are plotted as mean ± SEM; * indicates statistically significant differences (one-way ANOVA with Tukey's HSD post hoc test, *p* < 0.05). **D** Structure of the new triplex inhibitor VD5064. **E** Two thousand SC cells were seeded in 3D in type I collagen and incubated with CTX (3 μg/ml) alone or in combination with new generation of HGF/HGFL protease inhibitors MM3122 (25 μM) and VD5064 (25 μM) for 14–21 days. Colony counts are plotted as mean ± SEM; * indicates statistically significant differences (one-way ANOVA with Tukey’s HSD post hoc test, *p* < 0.05)

As outlined in Fig. 1A, HGF and HGF-like protein (HGFL) are synthesized as inactive single polypeptide precursors (or zymogens) and are secreted into the extracellular medium. HGF/HGFL pre-propeptides start with an N-terminal signal peptide (grey, removed co-translationally), followed by a plasminogen-apple-nematode (PAN) domain (purple), four kringle domains (orange), and a serine protease homology (SPH) domain (red) [18]. Inactive HGF/HGFL are converted into active forms by proteolytic cleavage, which generates an α-chain consisting of PAN and kringle domains and a β-chain that contains a SPH domain [19, 20]. The α and β chains in active HGF/HGFL are held together by a disulfide bond. The major proteases of HGF/HGFL are HGF activator (HGFA), Matriptase, and Hepsin [21–23]. We elected to inhibit these proteases by several small-molecule inhibitors that we have developed to test their effect on overcoming cetuximab resistance [24, 25].

For testing the HGF/HGFL protease inhibitors, we initially chose the CRC cell lines SC and CC-CR that exhibit de novo and acquired modes of cetuximab resistance, respectively, and both have elevated MET and RON phosphorylation levels and respond to the MET/RON inhibitor crizotinib [10–12]. MET/RON were not amplified or mutated in these lines. We initially tested the previously described “triplex” peptide-based, small molecule HGF/HGFL protease inhibitors—ZFH7116 and VD2173—in overcoming cetuximab resistance in SC and CC-CR cells [24]. ZFH7116, VD2173, or cetuximab alone were unable to significantly reduce colony number in 3D cultures of SC (Fig. 1B). However, both ZFH7116 and VD2173 in combination with cetuximab, markedly inhibited SC colony growth (Fig. 1B). Additionally, average colony area and total colony area of SC 3D colonies were also reduced in the cetuximab and ZFH7116 (Fig. S1A) or VD2173 (Fig. S1B) combinations. Similarly, in CC-CR cells, the combination of cetuximab with ZFH7116 or VD2173 showed cooperative reduction in colony number (Fig. 1C), average colony area, and total colony area (Fig. S1C, D). We then tested another protease inhibitor, MM3122, as well as a recently developed protease inhibitor, VD5064, whose structure is shared in Fig. 1D. Experimental details on the synthesis and initial characterization of VD5064 will be published separately elsewhere [26]. Both, MM3122 and VD5064 showed cooperative inhibition with cetuximab in terms of SC colony count (Fig. 1E), average colony area, and total colony area (Fig. S2A, B, C). Combined, these results indicate that both de novo and acquired cetuximab resistance associated with MET/RON hyperphosphorylation may be dependent on HGF/HGFL ligands and their proteolytic maturation.

### HGF overexpression imparts cetuximab resistance and leads to loss of 3D polarized phenotype

**Fig. 2:**
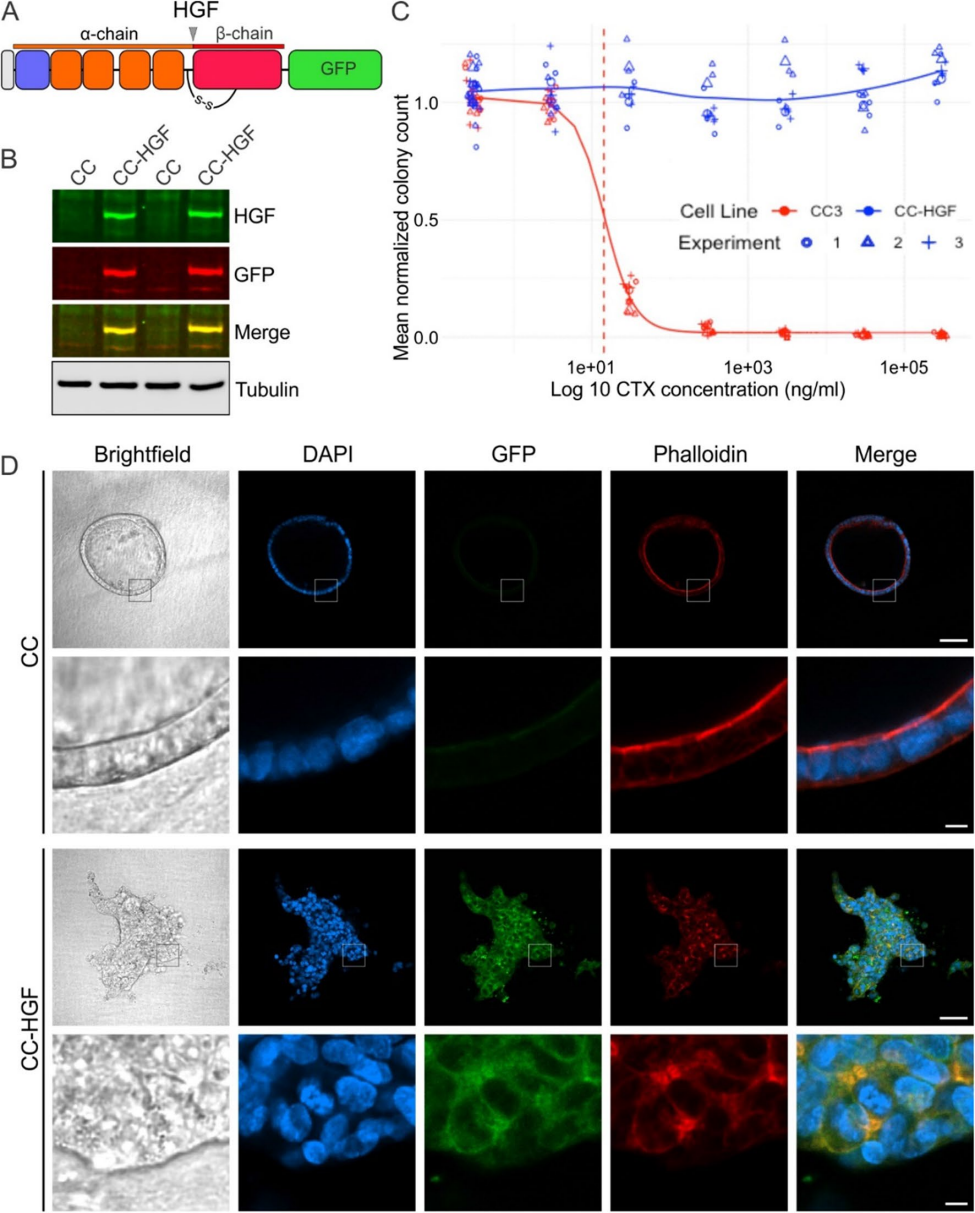
HGF overexpression imparts cetuximab resistance in CC cells. **A** Full-length human HGF was tagged with GFP at its carboxy terminus to engineer overexpression of HGF in cetuximab (CTX)-sensitive CC cells to generate HGF-overexpressing CC-HGF cells. **B** CC versus CC-HGF cells grown on plastic were lysed, resolved on SDS/PAGE, and immunoblotted for the indicated proteins. **C** CTX IC_50_ determination in 3D: Two thousand CC and CC-HGF cells were seeded in type I collagen and incubated with indicated CTX concentrations for 14 days. Colony counts normalized to untreated controls are plotted from three individual experiments performed in triplicate. **D** Brightfield and fluorescent confocal images of CC and CC-HGF cell colonies imaged through their equatorial plane stained for DNA (DAPI, blue), HGF-GFP (GFP, green), and F-actin (Phalloidin, red). (Scale bars main panels: 100 μm; insets: 10 μm)

Since receptor inhibition [10, 11] and ligand processing inhibition (Fig. 1) overcomes cetuximab resistance, we next sought to determine if autocrine ligand overexpression and endogenous maturation is *necessary and sufficient* to induce cetuximab resistance. We elected to overexpress GFP-tagged full-length human HGF in cetuximab-sensitive CC cells (Fig. 2A). CC-HGF cells thus derived showed overexpression of the chimeric protein compared to their parents by immunoblotting (Fig. 2B). We next compared cetuximab sensitivity of CC and CC-HGF cells in 3D (Fig. 2C). As expected, parental CC cells were extremely sensitive to cetuximab with an IC50 of 14.2 µg/ml. In contrast, the CTX IC50 for CC-HGF cells was indeterminable; there was no significant reduction in 3D colony counts even at the highest CTX concentration (Fig. 2C). A similar strategy for HGFL overexpression was unable to confer cetuximab resistance in CC cells and was not pursued further. Additionally, CC cells remained sensitive to cetuximab in conventional 2D plastic cultures, but CC-HGF cells were highly resistant to CTX with an indeterminable IC50 (Fig. S3).

We next tested for the effect of HGF overexpression on 3D colony morphology. Consistent with previous studies, most CC colonies exhibited a polarized cystic phenotype characterized by a hollow lumen surrounded by a single cell thick layer of cells with their apical surfaces facing inwards (Fig. 2D, upper CC panels). However, CC-HGF colonies lost their cystic appearance; they did not have a hollow lumen, were irregular, and had long projections reaching into the 3D matrix (Fig. 2D, lower CC-HGF panels). Thus CC-HGF colonies resembled a “spiky” phenotype instead, like previously observed in SC cultures, which are the cetuximab-resistant counterpart of CC cells [10]. Previous reports have shown that HGF is a potent inducer of an epithelial-to-mesenchymal phenotype (EMT), where nonmotile epithelial cells with apico-basolateral polarity adopt a migratory phenotype concomitant with loss of junctional integrity and apical surfaces [27, 28]. Combined, these results indicate that HGF overexpression is necessary and sufficient to induce loss of polarity and confer cetuximab resistance to a polarizing cetuximab-sensitive CRC line.

### HGF overexpression induces transcriptional reprogramming of cetuximab-sensitive cells

**Fig. 3:**
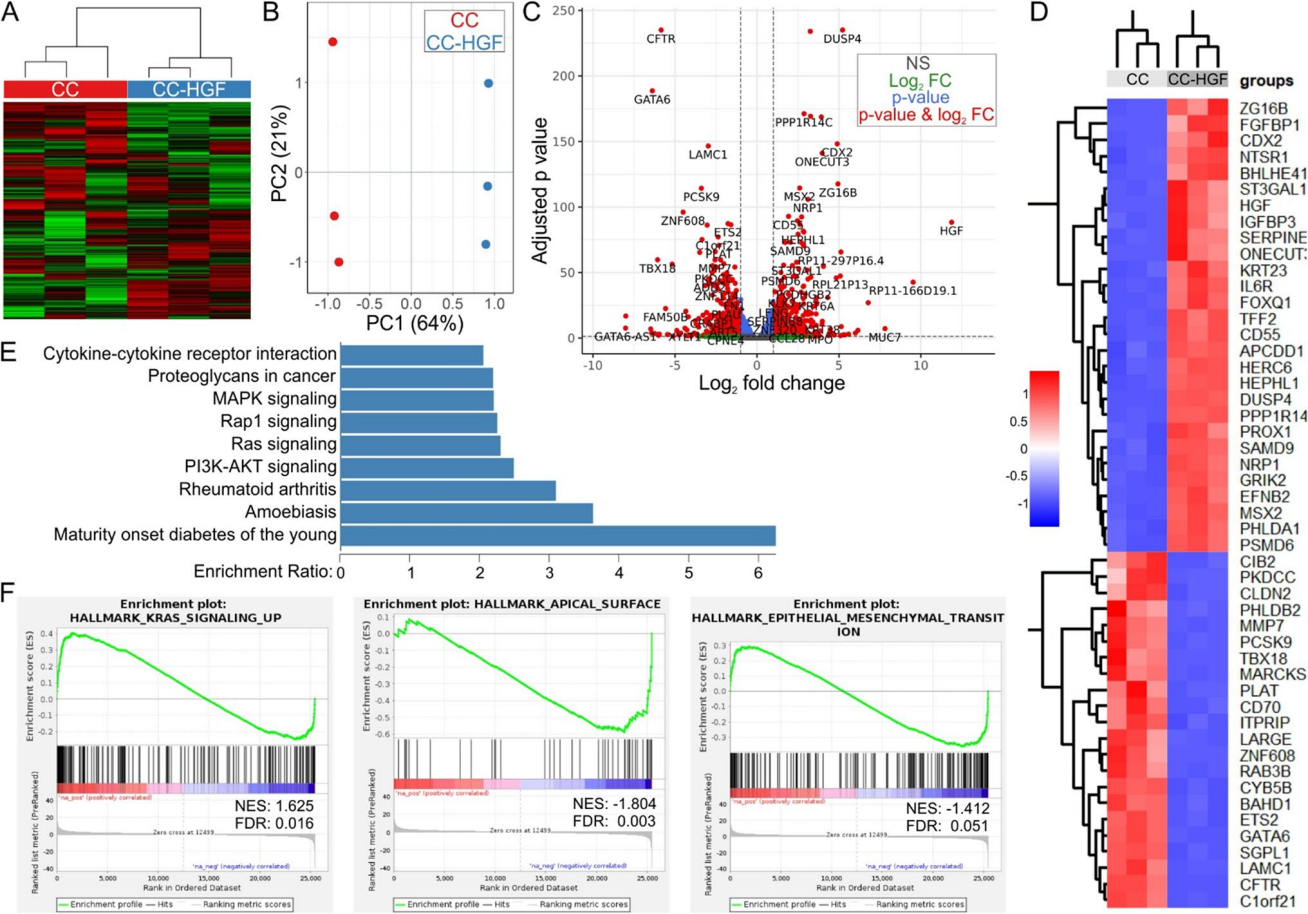
Transcriptional reprogramming of cetuximab-sensitive CC cells as a result of HGF overexpression. **A** All correlation heatmap using top 25% variant genes identified by RNA-seq data from CC and CC-HGF 3D cultures in three independent 3D cultures (type I collagen). **B** Principal component analysis (PCA) plot of RNA-seq data from CC and CC-HGF 3D cultures in triplicates. **C** Volcano plot for all comparisons all correlation PCA. Differential expression analysis criteria: absolute fold change ≥ 2 and FDR adjusted *p* value ≤ 0.05. **D** Heatmap of top 50 differentially expressed transcripts in CC vs CC-HGF 3D cultures in triplicates; red = up, blue = down (expression scale in inset). **E** WebGestalt-based CC vs CC-HGF pathway over-representation analysis. KEGG pathways significantly overrepresented (FDR ≤ 0.5) are depicted with their respective enrichment ratios. **F** Gene set enrichment analysis (GSEA) of RNA-Seq data from CC and CC-HGF cultures. Three select categories of interest are shown and a comprehensive list is shared in Supplementary Fig. S4. Abbreviations: NES = Normalized enrichment score; FDR = False discovery rate q-values

As shown in Fig. 2, HGF overexpression induced cetuximab resistance and morphologic changes in CC 3D cultures. To identify key pathways that may be modulated by HGF overexpression, we next compared gene expression (RNA sequencing, RNA-seq) of CC and CC-HGF cells grown in type I collagen 3D culture for two weeks. As shown in Fig. 3A, all correlation heatmap using top 25% variant genes was significantly different between CC and CC-HGF cells. RNA-seq principal component analysis also revealed that up to 64% (PC1) of variance could be attributed to HGF expression (CC vs CC-HGF), and up to 21% variance (PC2) was due to differences among replicates (Fig. 3B). We next performed a Volcano plot analysis to highlight genes that were significantly upregulated or downregulated in CC-HGF cells compared to parental CC cells (Fig. 3C). HGF (as expected) and DUSP4 were two of the top upregulated genes in CC-HGF, while CFTR and GATA6 were more enriched in CC cells. A heatmap of the top 50 differentially expressed transcripts is also shared in Fig. 3D. Next, we utilized the WEB-based GEne SeT AnaLysis Toolkit (WebGestalt) (Fig. 3E) and Gene Set Enrichment Analysis (GSEA) (Fig. 3F) to understand higher order functional information of pathways and processes modulated by HGF overexpression. WebGestalt analysis revealed that MAPK signaling was one of the most significantly upregulated pathways in CC-HGF cells (Fig. 3E). Similarly, GSEA showed upregulation of KRAS signaling in CC-HGF cells (Fig. 3F, left panel). Interestingly, hallmark apical surface (Fig. 3F, middle panel) and apical junctions (Fig. S4) were downregulated in CC-HGF cells, which is consistent with the loss of polarity and transformation to an EMT-like phenotype observed by microscopic analysis (Fig. 2D). Hallmark EMT signature, however, was not significantly enriched in CC-HGF cells (Fig. 3F, right panel), which might indicate a partial EMT, observed previously with HGF in 3D cultures [28]. A comprehensive list of hallmark pathways enriched or downregulated in CC-HGF cells is shared in Fig. S4.

### Downstream MET inhibition reverses cetuximab resistance and loss of polarity induced by HGF

**Fig. 4:**
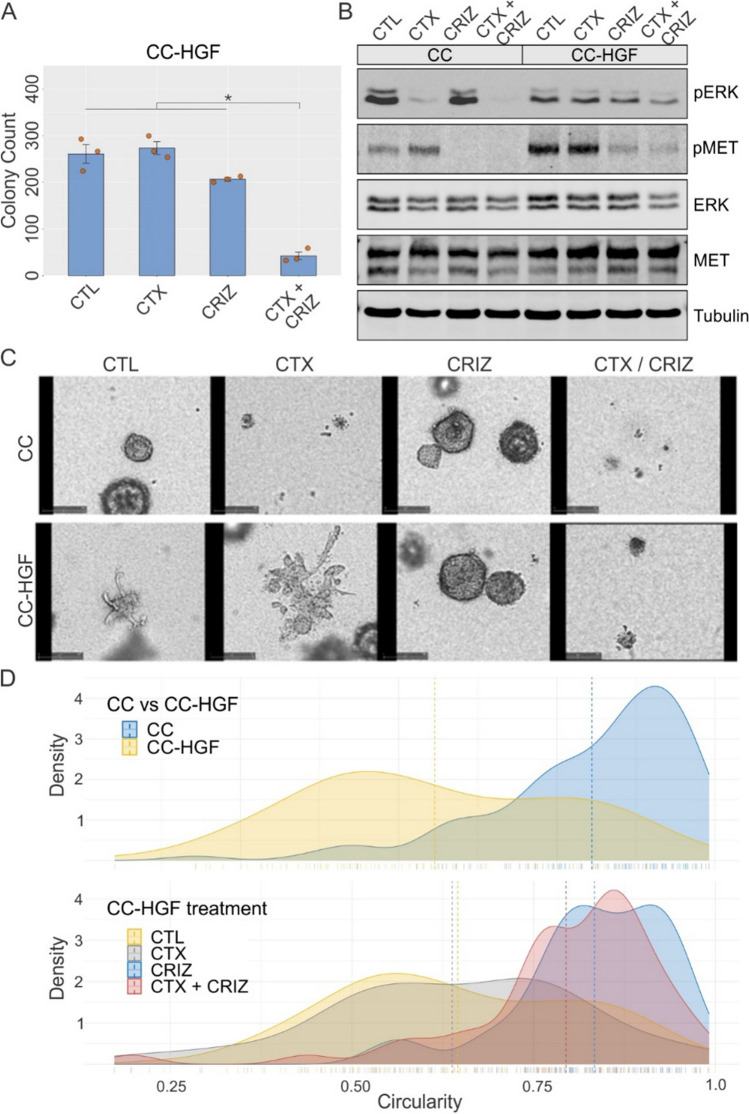
Crizotinib overcomes HGF-induced cetuximab resistance and loss of polarity. **A** Two thousand CC-HGF cells were seeded in type I collagen and incubated with cetuximab (CTX, 3 μg/ml) or crizotinib (CRIZ, 0.25 μM) for 14 days. Colony counts are plotted as mean ± SEM; * indicates statistically significant differences (one-way ANOVA with Tukey's HSD post hoc test, *p* < 0.05). **B** One hundred thousand CC and CC-HGF cells were cultured in type I collagen for seven days and incubated with CTX (3 μg/ml) and/or crizotinib (CRIZ, 0.25 μM) for an additional 48 h then lysed and resolved on SDS-PAGE followed by immunoblotting for proteins as indicated. **C** Live-cell imaging of CC vs CC-HGF treated with CTX and/or CRIZ: CC and CC-HGF cells imaged for 12 days with treatments indicated. Final image at the end of analysis is displayed. Supplementary movies contain the corresponding full-length movies of imaging of individual colonies under treatment. **D** Quantification of cystic and spiky morphology of cells grown in 3D for eight days with treatments indicated. Results are quantified as circularity index (see Methods); higher circularity index indicates rounder colonies and lower circularity index indicates spiky colonies. Top panel compares CC and CC-HGF circularity at baseline, and lower panel depicts CC-HGF cells under indicated treatment conditions. Dotted lines indicate median circularity index for indicated cells and treatment conditions

We next tested if induction of cetuximab resistance induced by upstream HGF overexpression can be compensated by downstream MET inhibition with the small molecule inhibitor crizotinib. Cetuximab or crizotinib alone were unable to significantly reduce colony number for CC-HGF cells in 3D cultures (Fig. 4A). However, both cetuximab and crizotinib combined, markedly inhibited CC-HGF colony growth (Fig. 4A). Additionally, the average colony area and total colony area of CC-HGF 3D colonies were also reduced in the cetuximab and crizotinib combination (Fig. S5). We next tested the effect of cetuximab and/or crizotinib treatments at the signaling level by immunoblotting. Here, as expected, cetuximab treatment alone reduced ERK1/2 phosphorylation in CC cells (Fig. 4B). However, in CC-HGF this reduction was effective only in the cetuximab/crizotinib combination. Consistent with the drug’s specificity, only crizotinib and not cetuximab was able to reduce MET phosphorylation. Total ERK and MET levels were comparable between the lines and among treatments; tubulin levels show equal loading (Fig. 4B). Cetuximab and crizotinib treatment also led to a modest but significant G1 cell cycle arrest (Fig. S6), which was consistent with the previous observation of SC and CC-CR cells arrested in G1 by the cetuximab/crizotinib combination [11]. Modest cell-cycle arrest might, in part, be due to the presence of already cleaved, active HGF prior to inhibitor addition, which could linger for the duration of the assay.

We next tested if crizotinib addition can reverse the loss of polarity phenotype in CC-HGF cells. For this we performed live imaging of CC and CC-HGF colonies under cetuximab and/or crizotinib treatment continuously for 12 days (Supplementary movie M1). Still images at the end of the imaging window are shared in Fig. 4C, which showed that CC colony growth was extremely sensitive to cetuximab but were unperturbed by crizotinib addition. In contrast, growth of CC-HGF cell colonies was not affected by cetuximab, but inhibition of colony growth was only observed in cetuximab/crizotinib combination. More interestingly, while crizotinib alone treatment didn’t affect colony growth of CC-HGF cells, it did change their morphology with the colonies appearing more cystic. The “spikiness” was quantified as a circularity index (see methods) and showed that CC-HGF colonies were spikier than CC colonies (Fig. 4D, upper panel). Next, CC-HGF colony spikiness was compared under cetuximab and/or crizotinib treatment and showed that crizotinib was able to re-circularize these colonies (Fig. 4D, lower panel). These results indicate that the loss of polarity and cetuximab resistance, though initiated by the same molecule (HGF), may be uncoupled by selective downstream modulation. Interestingly, a loss of polarity, which is associated with an EMT phenotype, is represented by the consensus molecular subtype 4 (CMS4) in CRC [29], and we found HGF overexpression to be highest in the CMS4 subtype (Fig. S7A). Finally, we added recombinant human HGF to another CRC line, HCT8, and observed a similar emergence of the spiky phenotype (Fig. S7B,C). Together, these results indicate that HGF-induced cetuximab resistance and loss of polarity can be overcome by downstream MET inhibition.

### HGF-induced cetuximab resistance can be overcome HGF protease inhibitors

**Fig. 5:**
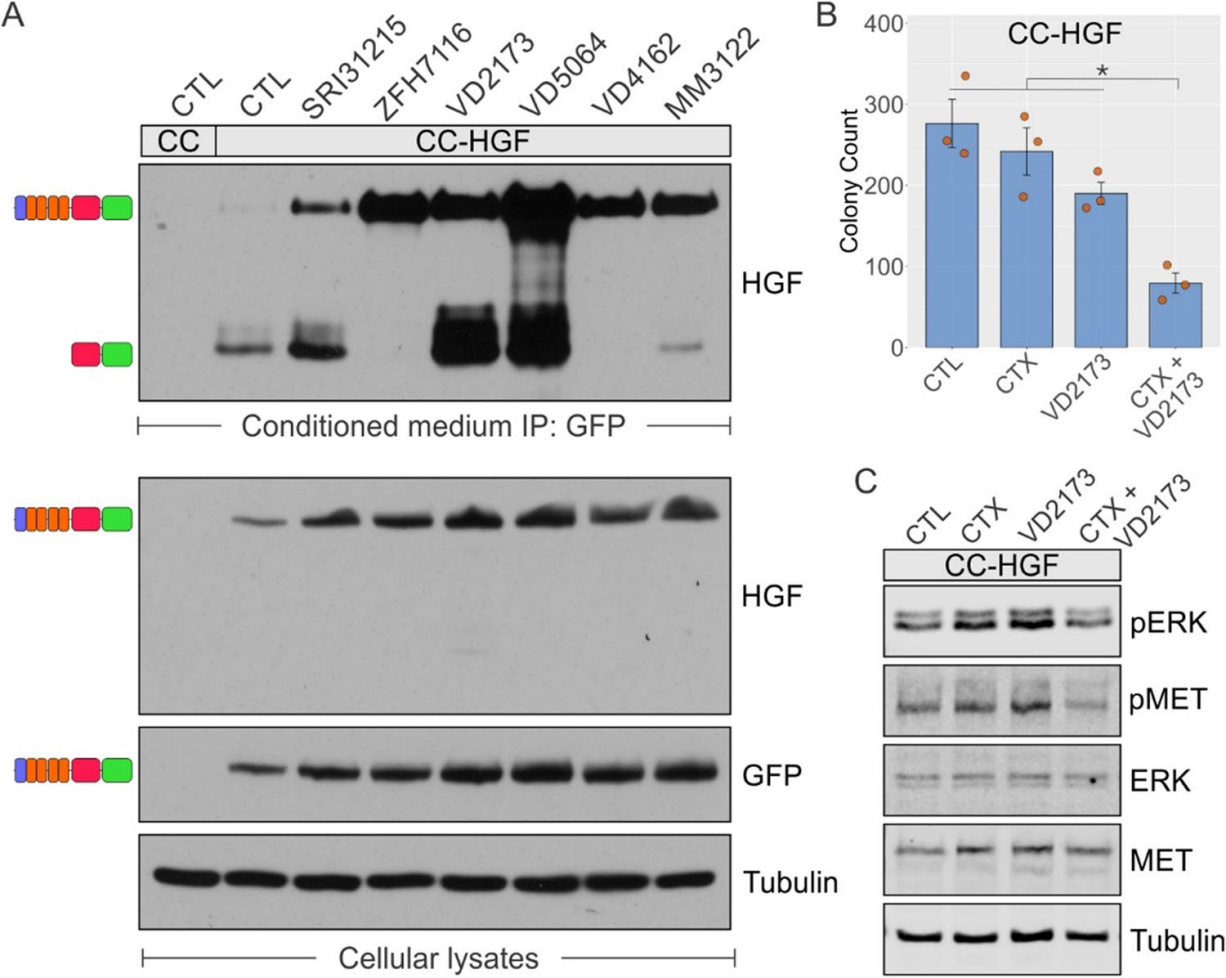
HGF protease inhibitors reduce HGF maturation and resensitize CC-HGF cells to cetuximab. **A** Two hundred thousand CC and CC-HGF cells were grown on plastic and four days later were incubated with inhibitors indicated (50 μM) for an additional 48 h. Conditioned media were then subjected to immunoprecipitation with GFP-Trap Magnetic beads and resolved on SDS-PAGE and probed with HGF antibody recognizing β-chain as indicated. Expected full-length (inactive) and cleaved GFP-bound βfragment (indicating protease activity) HGF isoforms are depicted as schematic on left of observed bands. Corresponding cellular lysates were immunoblotted for indicated proteins as depicted in lower panels. **B** Two thousand CC-HGF cells were seeded in type I collagen and incubated with cetuximab (CTX, 3 μg/ml) or VD2173 (25 μM) for 14 days. Colony counts are plotted as mean ± SEM; * indicates statistically significant differences (one-way ANOVA with Tukey's HSD post hoc test, *p* < 0.05). **C** One hundred thousand CC-HGF cells were cultured in type I collagen for seven days and incubated with CTX (3 μg/ml) and/or VD2173 (25 μM) for 48 h and then lysed and resolved on SDS-PAGE followed by immunoblotting for proteins as indicated

Here, we investigated if the HGF-GFP chimera followed the same biosynthesis and processing as endogenous HGF and if it could be a substrate for endogenous proteases. We anticipated that the full-length, inactive form HGF-GFP would be observed as a slower-migrating band on a Western blot, and the appearance of a faster-migrating band immunoreactive for HGF would indicate the cleaved, active form. For this, we tested the conditioned medium of CC-HGF cells incubated with several protease inhibitors [24, 25, 30]. All the protease inhibitors we tested showed accumulation of the full-length, uncleaved precursor form, which was more prominent with ZFH7116, VD2173, and VD5064 treatments (Fig. 5A, conditioned medium panel on top). Since HGF internalization and degradation is dependent on MET binding and subsequent lysosomal degradation, higher HGF levels were also observed with the protease inhibitors [31]. Finally, with some inhibitors, like VD2173 and VD5064, we also observed higher levels of the GFP-bound beta fragment, which could be a function of total HGF levels, or due to the action of other proteases unaffected by the inhibitor used. The inhibitors used also have different specificity to HGF proteases [24, 25]. Immunoblots from corresponding total lysates show equal loading (Tubulin) and HGF expression (GFP, HGF). We next tested if cetuximab resistance could be overcome by the potent protease inhibitor, VD2173, in CC-HGF cells. VD2173, in combination with cetuximab, markedly inhibited CC-HGF colony growth compared to controls or individual treatments (Fig. 5B). Additionally, the average colony area and total colony area of CC-HGF 3D colonies were also significantly reduced in the cetuximab and VD2173 combination (Fig. S8). At the signaling level, the cetuximab/VD2173 combination was more effective in reducing ERK1/2 phosphorylation levels compared to individual treatments (Fig. 5C). Combined, these results indicate that HGF-GFP is a biologically active molecule, and its cetuximab resistance phenotype can be overcome by inhibition of its processing.

### Positive and negative regulators of MET/RON pathway and their expression in TCGA CRC datasets

**Fig. 6:**
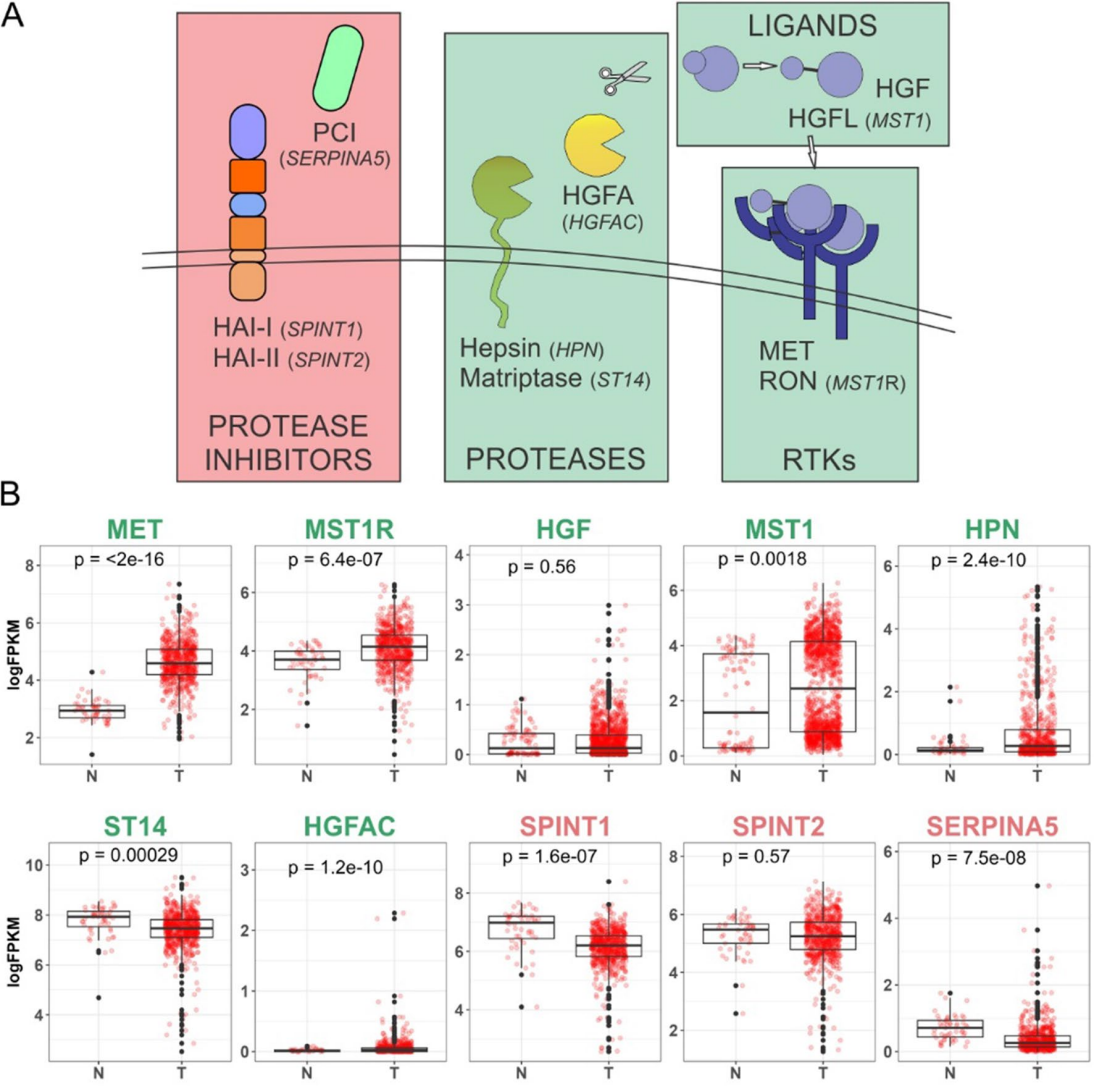
Positive and negative regulators of MET/RON pathway and their expression in TCGA CRC data. **A** Schematic of key signaling molecules in the MET/RON signaling pathway upstream of the receptor; positive regulators are boxed in green while negative regulators are boxed in red. **B** TCGA expression analysis of the ten MET/RON upstream pathway members; expression of individual genes as indicated in normal (N) colon compared with CRC tumors (T). Consistent with the schematic above, positive regulators of MET/RON signaling are labeled in green while negative regulators are labeled in red. P-values from T test comparison (Wilcoxon rank-sum test) between normal and tumor samples are included on top of each graph and *p* < 0.05 indicates statistically significant differences

In the Fig. 6A schematic, we depict the major upstream positive and negative regulators of MET/RON signaling and their functional relationship. Matriptase (*ST14*), hepsin (*HPN*), and HGFA (*HGFAC*) are serine proteases that contribute to the proteolytic processing of the growth factors HGF and HGFL (*MST1*). HGF and HGFL need to be cleaved to become biologically active and bind to their respective receptors, MET and RON (*MST1R*), to trigger downstream signaling activation, e.g., MAPK signaling. HAI-1 (*SPINT1*), HAI-2 (*SPINT2*), and Protein C inhibitor (PCI/*SERPINA5*) are endogenous serine protease inhibitors that have been shown to negatively regulate HGF/HGFL protease activity. These ten genes identified were further evaluated in the TCGA using colon and rectal carcinoma (COAD and READ) databases, where mRNA levels were compared between normal and tumor tissues (Fig. 6B). In our analysis, the receptors *MET* and *MST1R* displayed increased mRNA expression in tumor samples. *MST1*, the ligand for *MST1R*, was also elevated at the mRNA level in tumor samples. HGF failed to show significant upregulation in CRC overall but as mentioned earlier (Fig. S7A), HGF was higher in the CMS4 CRC subtype. Expression levels varied for the serine proteases with an increase in tumor mRNA levels for *HPN* and *HGFA*, while *ST14* was downregulated in tumor samples. All three negative regulators, the endogenous protease inhibitors, demonstrated a trend for reduced mRNA expression in tumor tissues compared to normal, with *SPINT1* and *SERPINA5* having a reduction in mRNA expression that reached statistically significant levels.

The small-molecule HGF processing inhibitors used in our study (ZFH7116, VD2173, MM3122, and VD5064) mimic the activity of the endogenous protease inhibitors. We have also observed the endogenous inhibitors of MET/RON signaling to be downregulated in de novo cetuximab resistant SC cells, while other positive regulators were relatively unperturbed, indicating that these negative regulators may be involved in cetuximab resistance (Fig. S9). While HAI-1/2 appear to only act on HGFA, PCI has a broader substrate specificity, and one of its substrates includes thrombin [32]. Since thrombin is an HGFA activator itself, depending on the context, PCI could both upregulate or downregulate MET/RON signaling [33, 34]. HAI-1 and HAI-2 are found to be widely expressed in epithelial cells [35]. Since there was no significant difference between normal and tumor tissue (Fig. 6B) for *SPINT2* (HAI-2), we decided to focus on *SPINT1*’s (HAI-1) contribution to cetuximab resistance.

### HAI-1 expression correlates with cetuximab resistance and HAI-1 addition overcomes HGF-induced cetuximab resistance

**Fig. 7:**
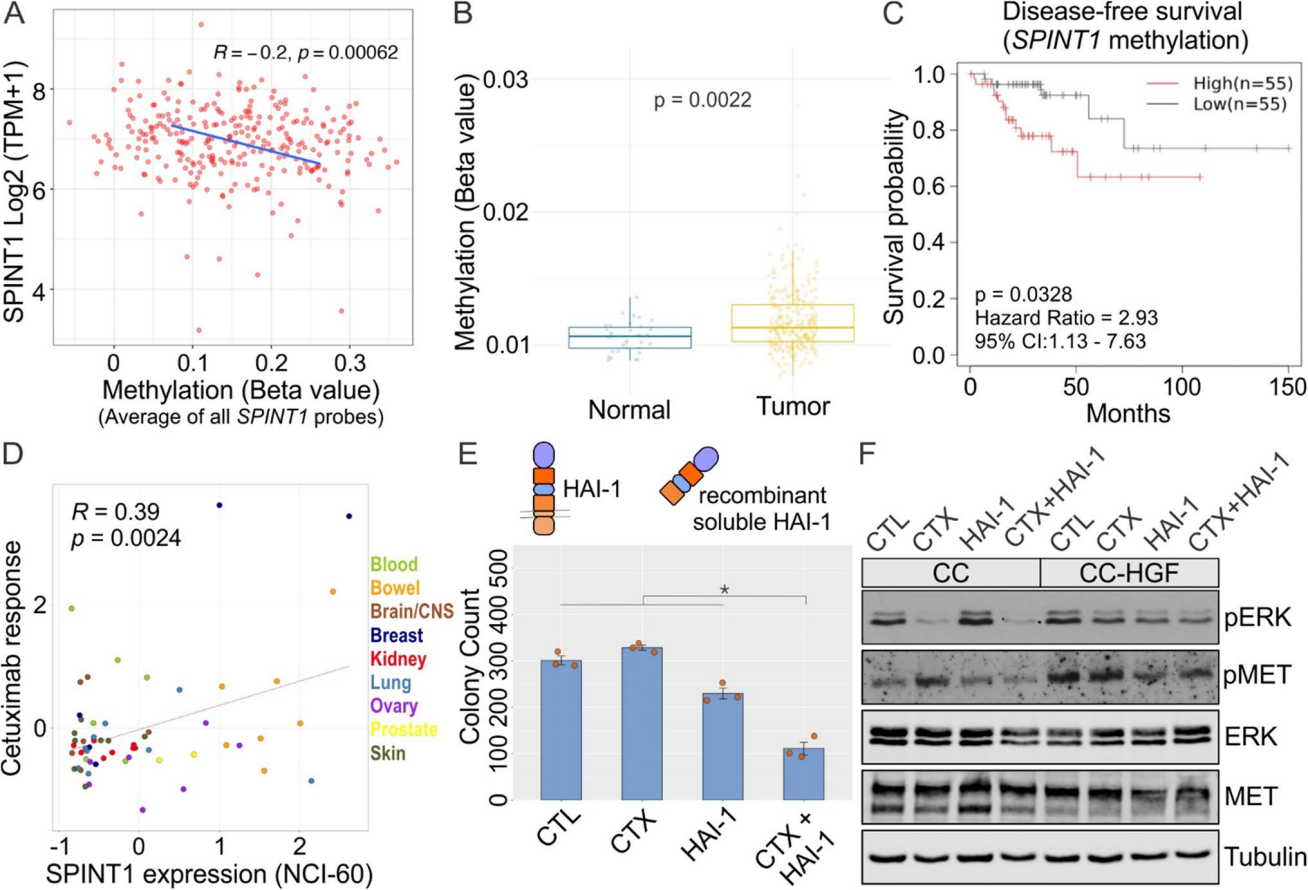
HAI-1 expression correlates with cetuximab sensitivity and HAI-1 addition overcomes HGF-induced cetuximab resistance. **A** Correlation analysis of *SPINT1* DNA methylation with its expression in CRC tumor samples. **B** *SPINT1* DNA methylation comparison between normal and tumor. **C** Survival analysis (disease-free survival) in TCGA database based on low vs high *SPINT1* DNA methylation. **D** Correlation between *SPINT1* expression and response to cetuximab (CTX) in NCI-60 cell lines. **E** Two thousand CC-HGF cells were seeded in type I collagen and incubated with CTX (3 μg/ml) or HAI1 (10 μg/ml) alone or in combination as indicated for 14 days. Colony counts are plotted as mean ± SEM. **F** One hundred thousand CC and CC-HGF cells were cultured in type I collagen for seven days and incubated with CTX (3 μg/ml) and/or HAI-1 (10 μg/ml) for 48 h and then lysed and resolved on SDS-PAGE followed by immunoblotting for proteins as indicated

HAI-1 (SPINT1), or hepatocyte growth factor activator inhibitor type I, is a type I membrane-bound serine protease inhibitor. HAI-1 binds to and inhibits HGFA and consequently inhibits HGF maturation [36]. To further assess the translational relevance of our findings in this study, we utilized the TCGA COAD database to examine the role of HAI-1 in CRC and cetuximab resistance. First, since *SPINT1* expression was low in CRC tissue compared to normal tissue, we tested if this difference in expression could be related to *SPINT1* DNA methylation. We observed that *SPINT1* genomic DNA methylation in CRC was higher than normal colon tissue (not shown). DNA hypermethylation is a mechanism of gene silencing that could result in decreased HAI-1 expression, and therefore, an increase in HGF processing. Further, *SPINT1* gene expression and methylation (average across all *SPINT1*-associated methylation probes) correlation analysis revealed a weak but positive correlation in colorectal cancer samples (Fig. 7A). Moreover, the promoter region of *SPINT1*, represented by the specific probe cg22491225, is hypermethylated in tumor samples compared to normal tissue (Fig. 7B). Additionally, we investigated the effect of *SPINT1* methylation on survival using the Shiny Methylation Analysis Resource Tool (SMART) [17]. Hypermethylation at probe cg22491225 showed a statistically significant (*p* = 0.0328) decrease in disease-free survival compared to hypomethylation with a hazard ratio of 2.93 (Fig. 7C). Two other nearby *SPINT1* promoter methylation probes—cg20647962 and cg27510007—also showed statistically significant methylation-specific disease-free survival in the TCGA COAD datasets.

Following our observations with the TCGA COAD database, we investigated the effect of HAI-1 on cetuximab resistance. CellMiner, a query tool for the NCI-60 cancer cell lines, was used to analyze the relationship between *SPINT1* expression and cetuximab sensitivity in a variety of cancer cell lines [37, 38]. Here, *SPINT1* expression had a significant positive correlation with cetuximab sensitivity, i.e., cetuximab sensitivity increased as *SPINT1* expression increased (Fig. 7D).

Based on the correlation of HAI-1 with CRC prognosis and cetuximab response in cancer databases and cell lines, we hypothesized that HAI-1 addition or overexpression would induce cetuximab sensitivity in HGF/MET-dependent cetuximab-resistant lines. To test this hypothesis, CC-HGF cells were seeded in 3D in type I collagen and treated with cetuximab and/or recombinant soluble human HAI-1. After 14 days, the combination treatment of cetuximab and HAI-1 significantly reduced CC-HGF colony count (Fig. 7E). Additionally, the average and total areas of CC-HGF colonies were also considerably smaller in the double treatment (Fig. S10). At the signaling level, CC cells exhibited a reduction in ERK1/2 phosphorylation levels with cetuximab treatment alone. However, this reduction was only effective after addition of HAI-1 in CC-HGF (Fig. 7F).

## Discussion

We previously reported that MET/RON hyperactivation—in the absence of gene amplification or mutation—contributes to cetuximab resistance in CRC. In this report, we have elucidated the mechanism of resistance—through autocrine secretion and activation of HGF that then activate MET. By selective manipulation of regulators upstream of MET, we show that HGF overexpression and its maturation is necessary and sufficient to induce cetuximab resistance and loss of polarity. We also show that HGF secretion and maturation is cell autonomous and that novel HGF protease small-molecule inhibitors are able to overcome de novo and acquired cetuximab resistance (novel compound VD5064, structure shared). Our studies also identify a role for the cellular HGF/HGFL protease inhibitor, HAI-1, in cetuximab resistance. We show that HAI-1 is downregulated in CRC by DNA hypermethylation, and its downregulation correlates with poor prognosis, particularly for the CMS4 subtype. More importantly, HAI-1 expression correlates with cetuximab response, and exogenous HAI-1 addition overcomes cetuximab resistance. Our molecular and pharmacological dissection of the MET/RON axis led to the identification of novel vulnerabilities to cetuximab resistance. These findings contrast with the generally accepted mode of HGF acting as a paracrine ligand.

### 3D type I collagen cultures

All of our studies here employed 3D cultures that were pioneered by Mina Bissell and Joan Brugge to grow breast epithelial cells in Matrigel, a basement membrane extract from a cancer cell line [39]. Subsequently, it was adopted rapidly by colon researchers like Alan Hall to understand biological principles of epithelial polarity [40]. Our early adoption and refinement of type I collagen cultures, however, highlights the advantages of the completely defined 3D matrix over Matrigel, which remains poorly defined and has high batch-to-batch variability. Using this system, we have previously identified and generated several modes of de novo and acquired cetuximab resistance in CRC lines [10–12]. Other investigators have adopted type I collagen cultures for colonic epithelial homeostasis and repair as well as for CRC and drug resistance studies [41–43]. One of the perceived limitations of these 3D culture systems is that they contain only one cell type, however, it can be mitigated by adding other cell types like stromal and immune cells to study cell nonautonomous effects and cell–cell interactions. As such, it remains a highly adaptable, powerful system to study CRC and understand and counter drug resistance.

### HGF in CRC cetuximab resistance

HGF overexpression has been implicated in a cetuximab-resistant CRC line, RKO [44]. High serum HGF levels have also recently been associated with poor prognosis of individuals with CRC undergoing cetuximab + FOLFIRI treatment [45]. However, these reports only probe for levels of HGF and not its activity. Our results highlight the critical regulatory step of HGF maturation that is achieved by the availability of active proteases. We further show that HAI-1, an endogenous inhibitor of these proteases, correlates with better prognosis and cetuximab response. Finally, since our studies included only cancer cells, we show that autocrine signaling by HGF—a widely recognized paracrine growth factor—can be a potent inducer of cetuximab-resistant phenotype.

### Genes and pathways modulated by HGF expression

HGF expression alone in cetuximab-sensitive cells initiated a series of signaling events that may further contribute to CRC progression and/or cetuximab resistance. Overexpression of the related RON ligand, HGFL, did not confer cetuximab resistance in CC cells, thus it was not pursued further. As expected, HGF overexpression induced MAPK and PI3K signaling that may confer a proliferation and survival advantage to cancer cells. The loss of polarity phenotype may additionally help cancer cell migration. In addition to the MET/RON axis, we noted other proteins like DUSP4 were high in CC-HGF cells. DUSP4 is one of 10 MAPK phosphatases, and several have been implicated in drug resistance [46]. DUSP4 specifically is associated with increased resistance to targeted therapy against another EGFR family member, HER2, in breast cancer [47]. In CRC, DUSP4 is one of five genes that was identified as a potential target to overcome CTX resistance [48]. Moreover, in a cetuximab sensitivity gene signature in CRC, DUSP4 is negatively correlated with cetuximab response [49]. Next, in cetuximab-sensitive CC cells, CFTR and GATA6 were two prominently overexpressed genes. We have previously shown that GATA6 is a negative regulator of WNT signaling and is involved in cetuximab resistance via downregulation of the long non-coding RNA MIR100HG and embedded miRNAs, miR-100 and miR-125b [12]. Thus, overexpression of GATA6 in cetuximab-sensitive lines is consistent with its role in cetuximab resistance. These findings also suggest a mechanistic link between WNT and RTK signaling and may, in part, explain why cetuximab-resistant CC-CR cells with high MIR100HG levels respond to both WNT inhibition and MET/RON inhibition [11, 12]. Corroborating our observation, CFTR expression also correlated positively with the CRC cetuximab sensitivity score [49]. Interestingly, CFTR is a target gene for mir-125b [50]. Furthermore, EGFR/CFTR pathways may negatively regulate each other; EGFR/ERK signaling modulates CFTR trafficking and induces degradation [51], while CFTR negatively regulates EGFR levels [52]. Thus, CFTR/EGFR crosstalk warrants further investigation. As such, our system remains suitable to study drug resistance and perform mechanistic studies.

### HGF in epithelial polarity

A striking phenotype we observed upon stably overexpressing HGF in CC cells was the loss of polarized structures of colonies in 3D culture. CC colonies are typically round with hollow lumens. In marked contrast, CC-HGF colonies formed protrusions into the matrix and lacked a lumen. A colony lumen, devoid of ECM for cells to attach, is typically an apoptotic environment, and suggests that HGF overexpression might confer a pro-survival advantage by activation of the MAPK and PI3K/AKT pathways. HGF is also known as scatter factor and has been shown to promote motility and matrix invasion of epithelial cells [53]. At the signaling level, HGF overexpression also led to the downregulation of apical junction and apical surface pathways, indicating a loss of polarity and an EMT-like phenotype. HGF thus induced both matrix invasion, loss of polarity, and cetuximab resistance. This is consistent with previous reports, where HGF exogenous addition also led to decreased junctional integrity of colonic epithelial monolayers [54]. HGF promoted epithelial spreading and RhoA GTPase activation, in part, by increased paxillin levels [55]. Interestingly, MET has been described as a basolateral protein, but whether the secretion of its ligand HGF is also polarized, remains to be determined [54].

In CRC molecular subtyping, CMS 1–4 subtypes correspond to immunological, canonical, metabolic, and mesenchymal phenotypes, respectively [29]. Loss of polarity and an EMT-like phenotype is enriched in the CMS4 subtype of CRC, and HGF overexpression was highest in the CMS4 subtype. In CC-HGF cells, HGF-induced loss of polarity and matrix invasion could be countered by downstream MET inhibition with crizotinib, confirming a functional link. Hallmark EMT signature, however, was not significantly enriched in CC-HGF cells (Fig. 3F, right panel), which might indicate a partial EMT, observed previously with HGF in 3D cultures [28].

### Small-molecule triplex HGF/HGFL protease inhibitors

Triplex inhibitors used in this study mimic HAI-1/2 activity and inhibit all three HGF/HGFL proteases HGFA, Matriptase, and Hepsin. These peptide-based inhibitors were initially synthesized as linear molecules like SRI31215 and ZFH7116 [30, 56]. Newer iterations like VD2173 and MM3122, were designed as circular molecules that have improved pharmacodynamic properties and longer half-life compared to their linear predecessors [24, 25]. MM3122 is additionally an excellent TMPRSS2 inhibitor and hence could be a promising candidate against COVID-19 [25]. Likewise, VD5064 is a cyclic peptide-based inhibitor with excellent pharmacodynamics and half-life that inhibits all three HGF/HGFL proteases.

### HAI-1 and other HGF protease inhibitors in CRC and cetuximab resistance

Our studies highlight a novel role of HAI-1 in CRC cetuximab resistance. HAI-1 knockout mice have previously been shown to develop intestinal tumors, illuminating HAI-1’s role as a tumor suppressor, but its role in cetuximab resistance was unknown [57]. HAI-1 expression was down in CRC and more significantly in the CMS4 subtype, consistent with HGF upregulation in that subtype (Fig. S10A). Our identification of the role of HAI-1 and its DNA hypermethylation in CRC prognosis and cetuximab resistance is novel and translationally relevant. Finally, HAI-1 is a transmembrane protein, but it may also be processed into a soluble form, which we used in this study to overcome cetuximab resistance. HAI-1 cleavage has been reported previously and may be mediated by metalloproteases [58]. Thus, HAI-1 trafficking, cell-surface delivery, and proteolytic processing warrant further in-depth investigation. We have previously identified that the polarized trafficking of transmembrane and secreted proteins is critical for the pathways they regulate, which can get dysregulated in cancer [59–61]. HAI-2 subcellular localization, for example, modulates its protease inhibitor activity [57, 62]. Finally, all three HGF protease inhibitors, HAI-1, HAI-2, and PCI may regulate the MET/RON axis in different CRC contexts. We highlighted the role and regulation of HAI-1/*SPINT1* in cetuximab resistance here, but we also identified HAI-2/*SPINT2* in the cetuximab sensitivity score discussed above [48]. Additionally, PCI was the most downregulated of the three in our cetuximab-resistant SC cells (Fig. S9).

### Study limitations

This is a more mechanistic, in vitro study, and further studies are needed focusing on the translational implications of these findings using in vivo models or in CRC patient material. The effect of the combined EGFR/MET inhibition on normal colonic epithelial cells and cell or tissue systems at other sites (e.g., liver, kidneys, lungs) where these pathways are needed hasn’t been studied either [63]. The new inhibitors shared, also need further follow-up with pharmacologic, bioavailability, and efficacy studies. Further studies of *SPINT1* promoter methylation and its expression are needed, as they do not necessarily correlate with each other [64]. Finally, while SPINT1 regulation by its CpG island methylation is interesting, more in-depth studies are needed to confirm its role in CRC prognosis and/or cetuximab resistance.

**Fig. 8:**
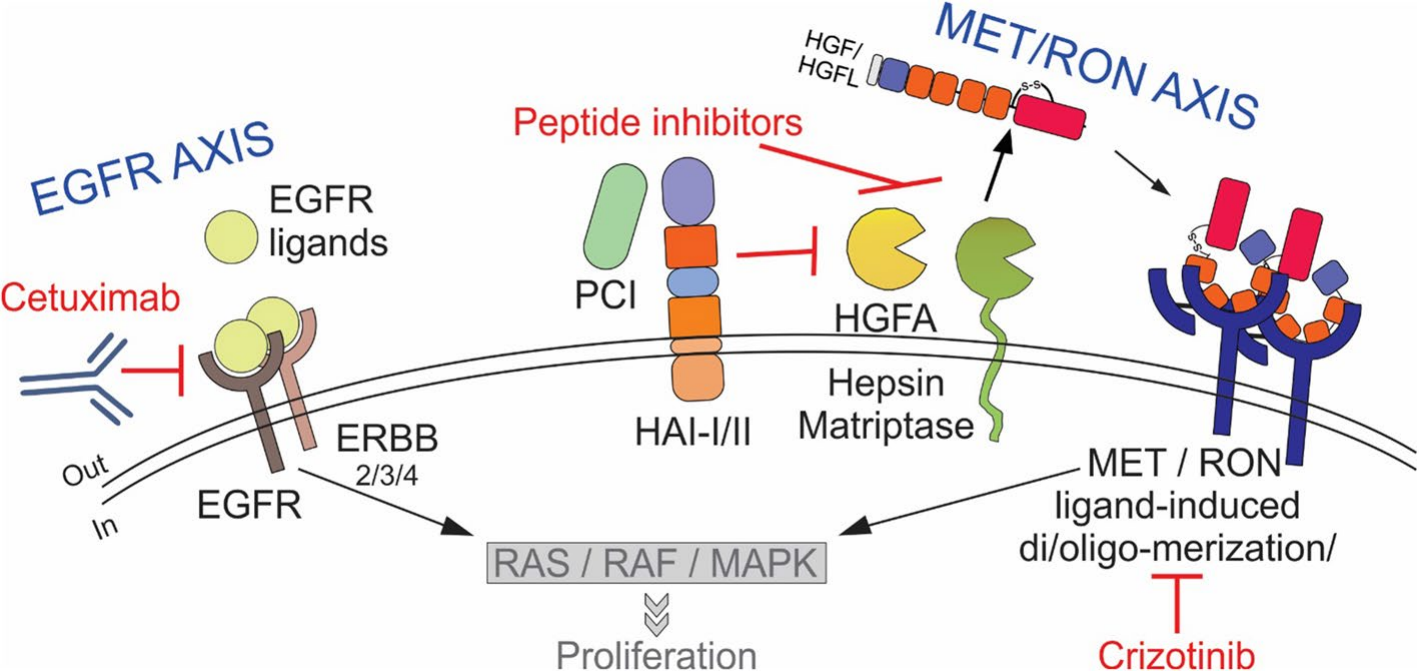
Schematic illustration of EGFR axis and MET/RON axis crosstalk in cetuximab resistance in CRC. Active EGFR and associated downstream signaling pathways confer several advantages to CRC cells including proliferation and/or evading apoptosis. Cetuximab counters EGFR signaling, but in cetuximab resistance, the shared downstream signaling pathways may be activated by other RTKs like MET/RON. MET/RON signaling may be inhibited at the receptor level by crizotinib, or at the ligand maturation level by exogenous inhibition of HGF/HGFL proteases HGFA, Hepsin, and Matriptase, or by endogenous protease inhibitors, PCI, HAI-I, and HAI-II. Inhibition of HGF/HGFL maturation may thus be a new strategy to combine with cetuximab inhibition in CRC

Collectively, our studies highlight the role of autocrine HGF expression and maturation, and the role of endogenous cellular proteases, and their inhibitors play in mediating cetuximab resistance. We also identified the prevalence of this pathway in CRC and its predictive power in cetuximab resistance. We further revealed nodes where this pathway may be inhibited to achieve synergy through combined inhibition of EGFR axis and MET/RON axis (schematic in Fig. 8). Finally, traditionally considered a paracrine factor, HGF’s autocrine role is increasingly being highlighted in several cancers, including acute myeloid leukemia, prostate cancer, lymphoma, and glioblastoma [65–68].

### Supplementary Information

Below is the link to the electronic supplementary material.

Supplementary file1 (PDF 1451 KB)

Supplementary file2 (MP4 33469 KB)

Supplementary file3 (CSV 249 KB)

## Data Availability

All data generated or analyzed during this study are included in this published article [and its supplementary information files]. All material generated during this research and detailed protocols are available upon request.
