# Supplementary material for: Inhibition of autocrine HGF maturation overcomes cetuximab resistance in colorectal cancer
Source: Cell Mol Life Sci. 2024 Jan 12;81(1):28. doi: 10.1007/s00018-023-05071-5 (PMC10784391; doi:10.1007/s00018-023-05071-5)
Supplement: Supplementary file 1 — Supplementary file1 (PDF 1451 KB) [file 18_2023_5071_MOESM1_ESM.pdf]

## SUPPLEMENTARY FIGURES

**Inhibition of autocrine HGF maturation overcomes cetuximab resistance in colorectal cancer**

Vivian Truong Jones[1,2]*, Ramona Graves-Deal[1,]*, Zheng Cao[1], Galina Bogatcheva[1], Marisol A Ramirez[3,4], Sarah J Harmych[1,5], James N Higginbotham[1], Vineeta Sharma[1], Vishnu C Damalanka[6], Claudia C Wahoski[1,7], Neeraj Joshi[1], Maria Johnson Irudayam[1], Joseph T Roland[8], Gregory D Ayers[3], Qi Liu[3,4], Robert J Coffey[1,9], James W Janetka[6], and Bhuminder Singh[1,9]

[1]Department of Medicine, Vanderbilt University Medical Center, Nashville, TN 37232, USA. [2]Department of Pharmacology, Vanderbilt University, Nashville, TN 37232, USA. [3]Department of Biostatistics, Vanderbilt University Medical Center, Nashville, TN 37232, USA. [4]Center for Quantitative Sciences, Vanderbilt University Medical Center, Nashville, TN, USA. [5]Department of Cell and Developmental Biology, Vanderbilt University, Nashville, TN 37232, USA. [6]Department of Biochemistry and Molecular Biophysics, Washington University School of Medicine, Saint Louis, MO 63110 USA. [7]Program in Cancer Biology, Vanderbilt University, Nashville, TN 37232, USA. [8]Department of Surgery, Vanderbilt University Medical Center, Nashville, TN 37232, USA. [9]Epithelial Biology Center, Vanderbilt University Medical Center, Nashville, TN 37232, USA.

*Co-first authors

[#]Corresponding Author:
Bhuminder Singh, PhD
2213 Garland Avenue
10465J, MRB IV
Vanderbilt University Medical Center
E-mail: bhuminder.singh@vumc.org
Nashville, TN 37232-0441

## SUPPLEMENTARY FIGURES

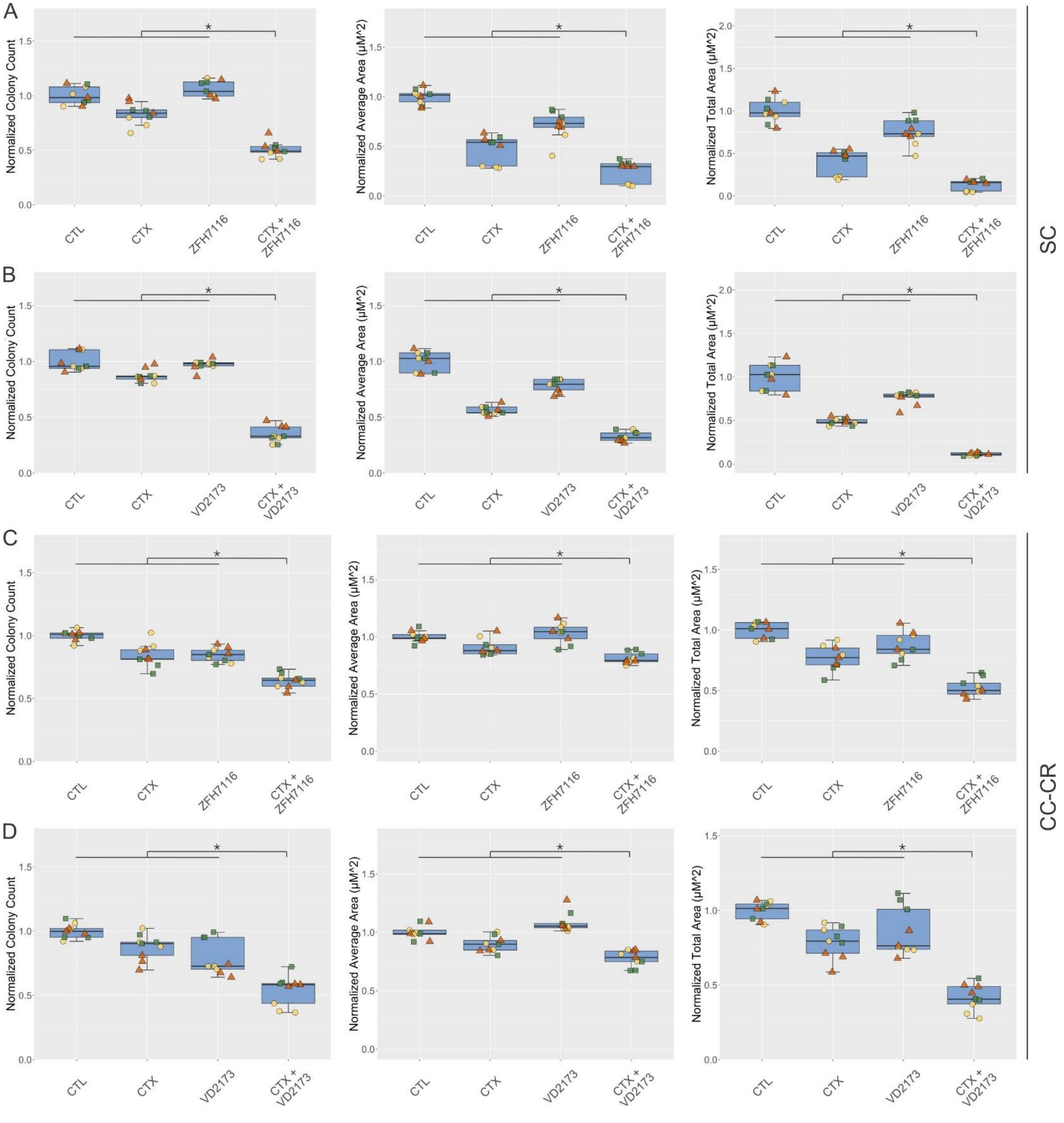


**Fig. S1. Quantification of HGF/HGFL protease inhibitors (ZFH7116 and VD2173) on overcoming *de novo* and acquired CRC cetuximab resistance in 3D collagen cultures. (A,B)** Two thousand SC and **(C,D)** CC-CR cells were seeded in 3D in type-I collagen and incubated with cetuximab (CTX, 3 μg/ml) alone or in combination with HGF/HGFL protease inhibitors ZFH7116 (50 μM) and VD2173 (50 μM), as indicated for 14-21 days. Three independent experiments performed in triplicate are plotted for normalized colony count (left panel), normalized average colony area (middle panel), and normalized total colony area (right panel). Values are plotted as mean ± SEM; * indicates statistically significant differences (one-way ANOVA with Tukey's HSD post hoc test, $p < 0.05$).

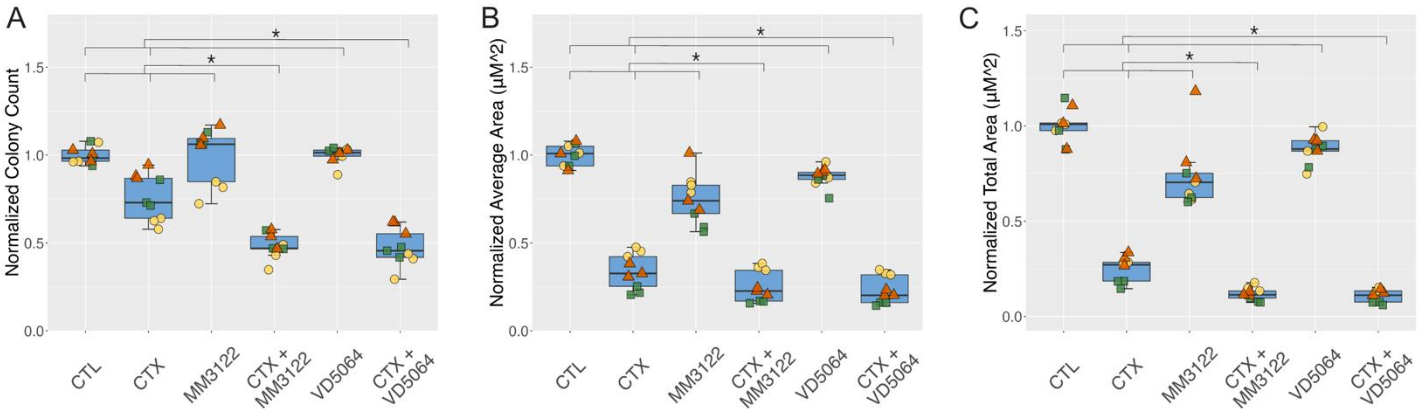


**Fig. S2. Quantification of new generation HGF/HGFL protease inhibitors (MM3122 and VD5064) on overcoming *de novo* and acquired CRC cetuximab resistance in 3D collagen cultures.** Two thousand SC cells were seeded in 3D in type-I collagen and incubated with cetuximab (CTX, 3 µg/ml) alone or in combination with HGF/HGFL protease inhibitors MM3122 (25 µM) and VD5064 (25 µM), as indicated for 14-21 days. Three independent experiments performed in triplicate are plotted for **(A)** normalized colony count, **(B)** normalized average colony area, and **(C)** normalized total colony area. Values are plotted as mean ± SEM; * indicates statistically significant differences (one-way ANOVA with Tukey's HSD post hoc test, $p < 0.05$).

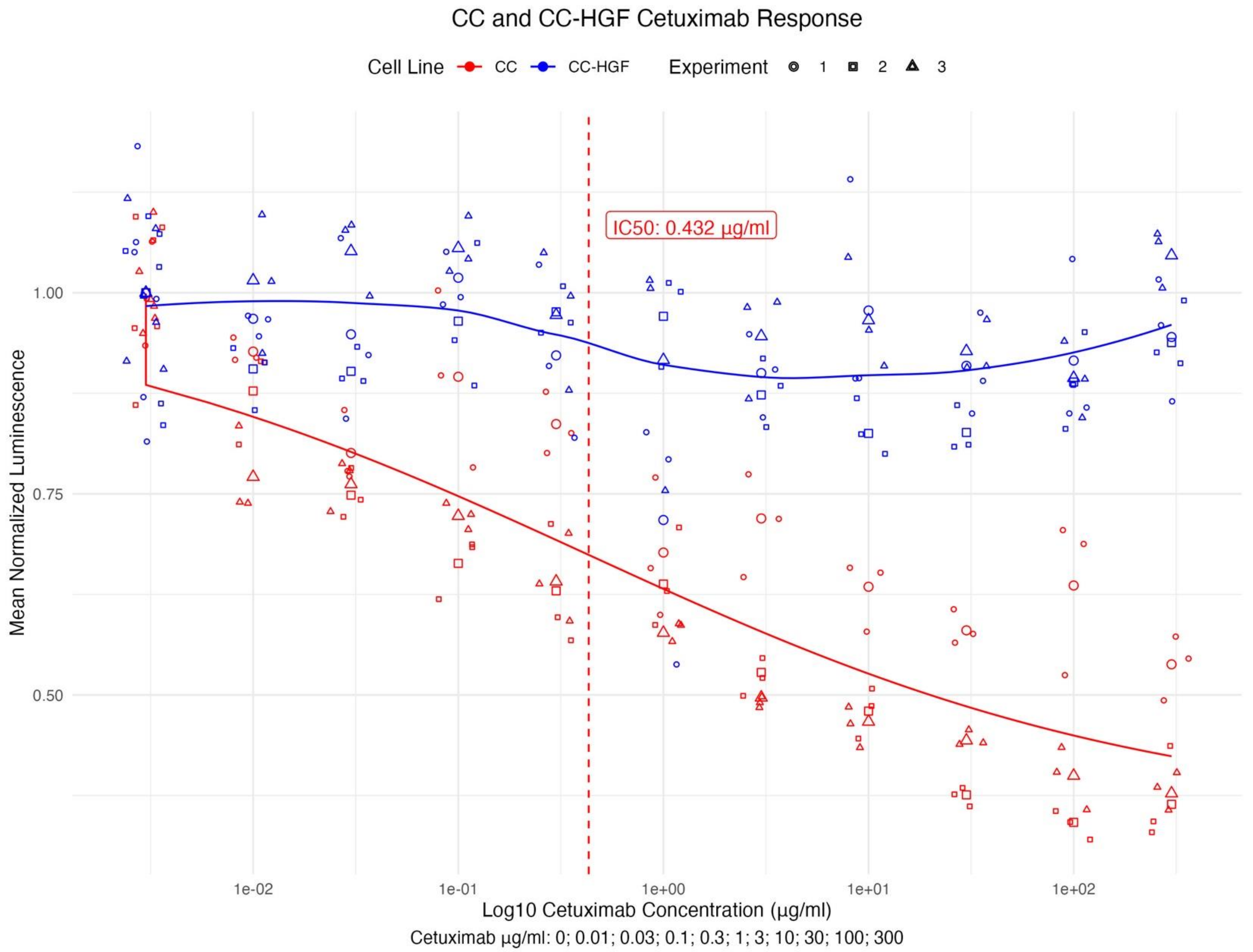


**Fig. S3. Cetuximab resistance determination for CC and CC-HGF cells in 2D.** Dose-response curves for cetuximab in 2D culture for CC (red) and CC-HGF (blue) cells were generated using CellTiter-Glo luminescence. Cells were seeded at 10,000 cells/well in a 96-well plate and treated for five days. The curve represents the mean of three independent experiments with three replicates each. The R package “drc” was utilized to determine IC50 values (Ritz et al., 2015).

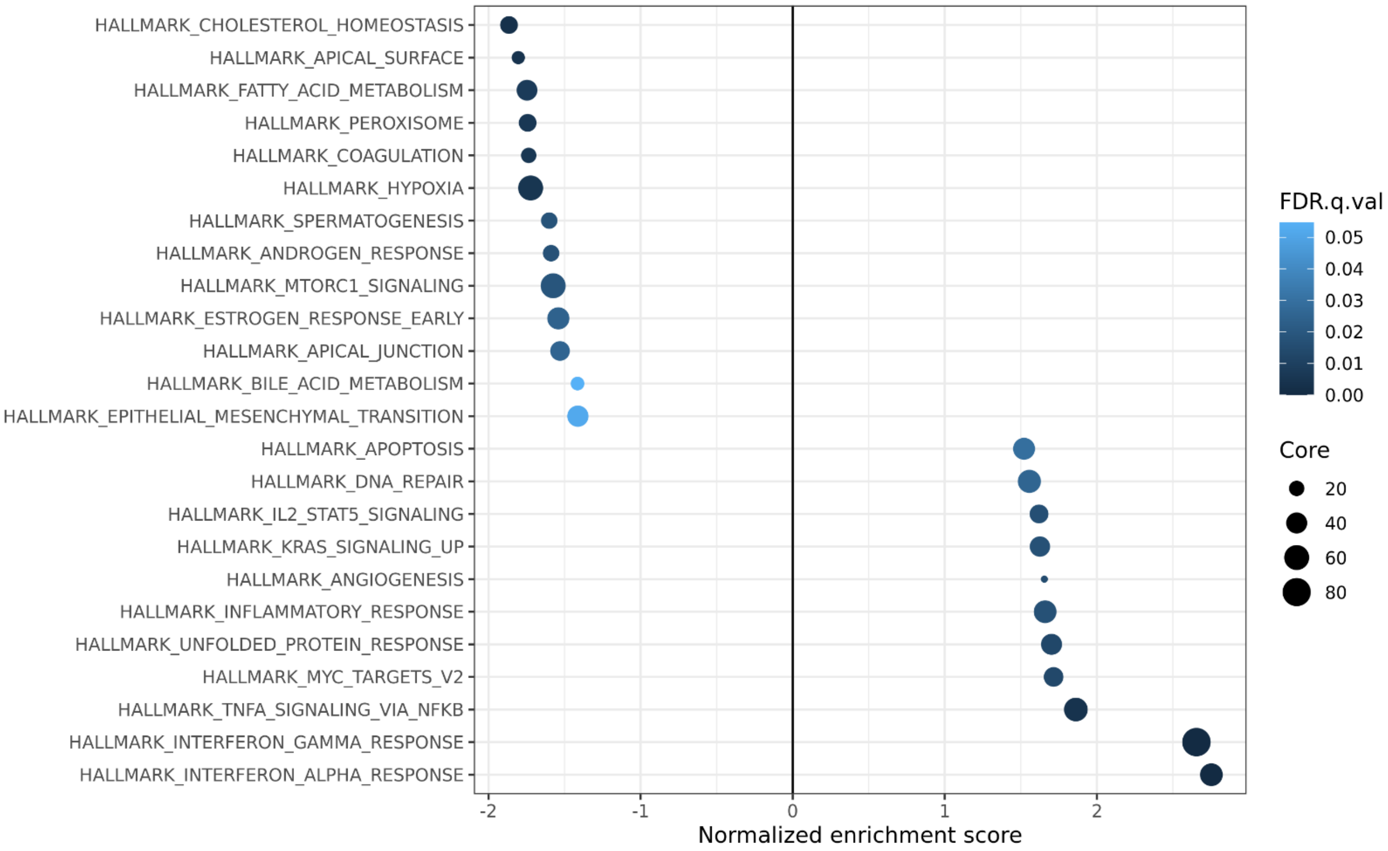


**Fig. S4. Hallmark gene set enrichment analysis comparison of RNA-seq data from CC and CC-HGF cells.** Hallmark pathways are indicated on the left and are represented as bubbles on the Normalized enrichment score on x-axis. Bubble size indicates core enrichment or number of leading-edge genes and bubble color represents FDR (false discovery rate); scales of both are indicated on the right.

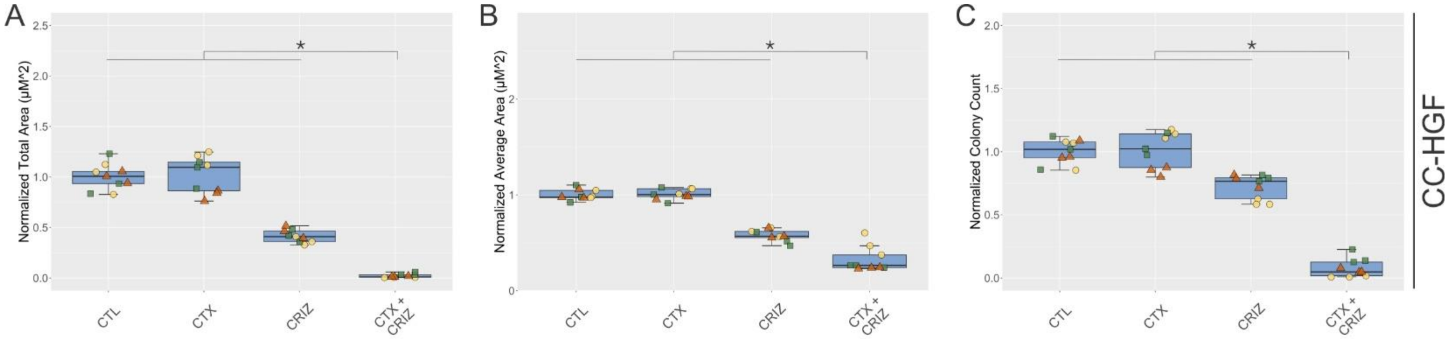


**Fig. S5. Quantification of CC-HGF 3D collagen cultures under treatment with cetuximab and crizotinib.** Two thousand CC-HGF cells were seeded in 3D in type I collagen and incubated with cetuximab (CTX, 3 μg/ml) alone or in combination with crizotinib (CRIZ, 0.25 μM) as indicated for 14 days. Three independent experiments containing three replicates each are plotted for **(A)** normalized colony count, **(B)** normalized average colony area, and **(C)** normalized total colony area. Values are plotted as mean ± SEM; * indicates statistically significant differences (one-way ANOVA with Tukey's HSD post hoc test, $p < 0.05$).

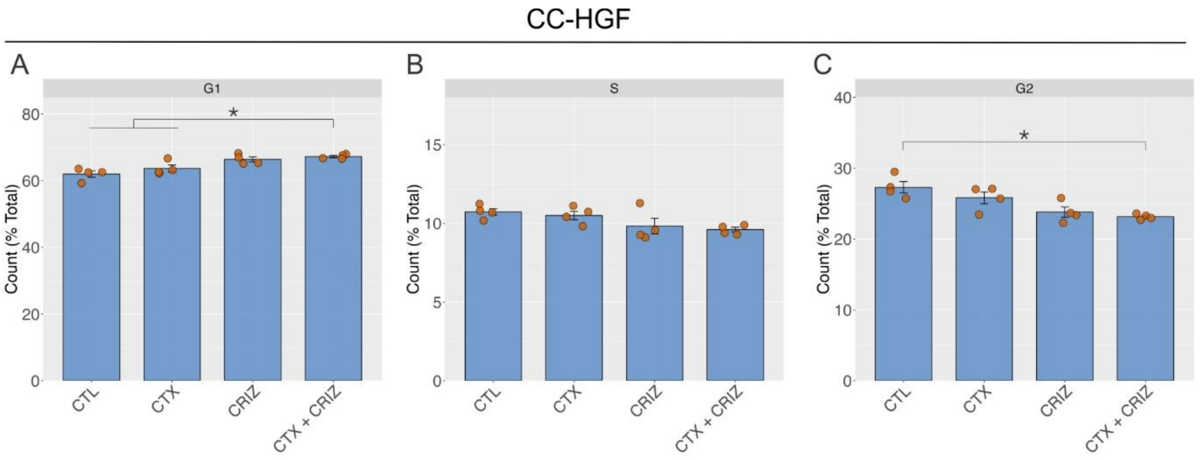


**Fig. S6. Cell Cycle analysis of CC-HGF cells.** One hundred thousand cells were grown in 3D culture for 7 days and then treated with cetuximab (CTX) and/or crizotinib (CRIZ) for two days. The middle collagen layers containing cells were separated from the outer collagen layers and then the cells were harvested and subjected to FACS analysis. Depiction of **A)** G1 phase, **B)** S phase, and **C)** G2 cell cycle phase distribution of CC-HGF cells with treatments indicated. Values are plotted as mean ± SEM; * indicates statistically significant differences (one-way ANOVA with Tukey's HSD post hoc test, $p < 0.05$).

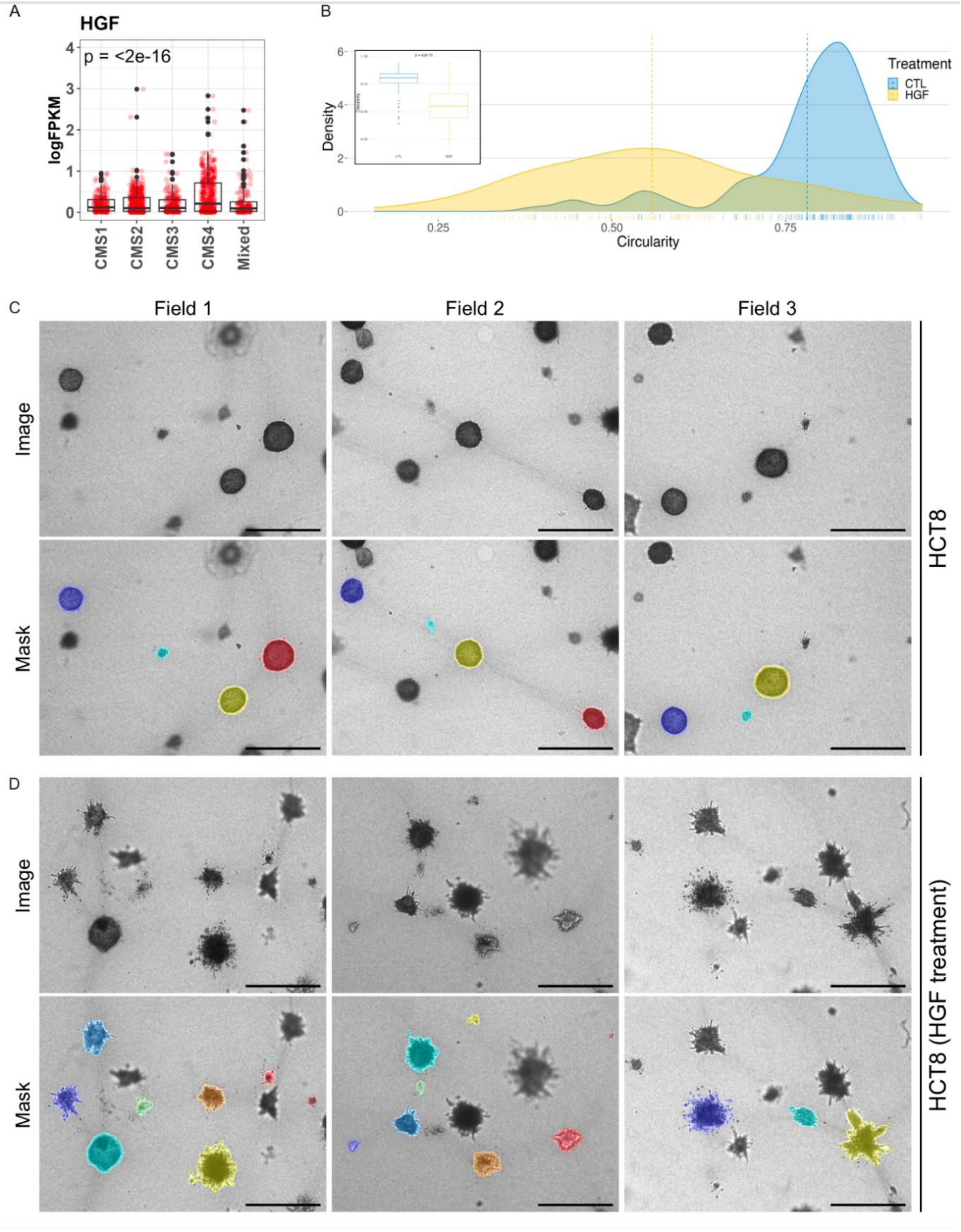


**Fig. S7. HGF expression in CRC subtypes and effect of HGF addition on HCT8 3D morphology. (A)** HGF expression in TCGA CRC datasets (CMS classification). **(B)** Two thousand HCT8 cells were seeded in 3D culture for 7 days to form cystic colonies. On day 7, cells were treated with 50 ng/ml recombinant human HGF. Images were captured after 4 days of HGF addition and colony morphology is depicted as circularity index as explained in the methods; higher circularity index indicates rounder colonies while spiky colonies have a low circularity index. Color-matched dotted lines indicate median circularity index for the treatment conditions. Inset also depicts median circularity and statistical significance (student's t-test, $p < 0.05$). **(C)** Three representative images from untreated HCT8 (upper panels) or HGF-treated HCT8 (lower panels) 3D cultures. Corresponding image masks are depicted under each individual image. Image masks identify only in-focus colonies based on

image texture and then draw colony margins. Images were analyzed using a combination of machine-learning and image analysis, as described in Methods.

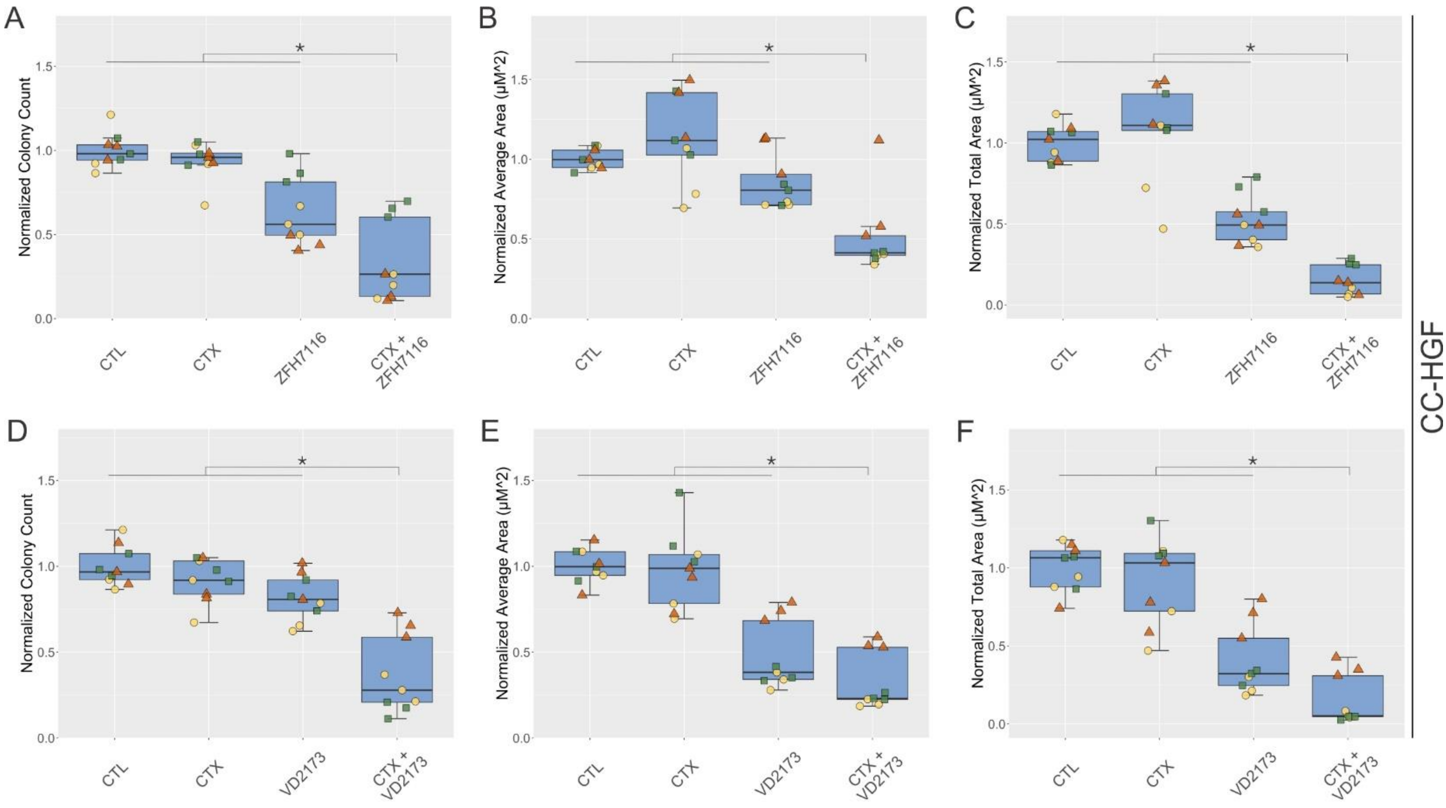


**Fig. S8. Quantification of CC-HGF 3D collagen cultures under treatment with cetuximab and protease inhibitors.** Two thousand CC-HGF cells were seeded in 3D in type-I collagen and incubated with cetuximab (CTX, 3 μg/ml) alone or in combination with VD2173 or ZFH7116 (50 μM) as indicated for 14 days. Three independent experiments containing three replicates each are plotted for **(A, D)** normalized colony count, **(B, E)** normalized average colony area, and **(C, F)** normalized total colony area. Values are plotted as mean ± SEM; * indicates statistically significant differences (one-way ANOVA with Tukey's HSD post hoc test, $p < 0.05$).

| Protein | Gene | Fold SC/CC |
|---|---|---|
| MET | MET | 1.14 |
| RON | MST1R | 1.13 |
| HGF | HGF | 0.97 |
| HGFL | MST1 | 1.01 |
| Matriptase | ST14 | 0.84 |
| HGFA | HGFAC | 1.06 |
| Hepsin | HPN | 1.07 |
| HAI-1 | SPINT1 | 0.98 |
| HAI-2 | SPINT2 | 0.81 |
| PCI | SERPINA5 | 0.61 |

**Fig. S9. CC versus SC comparison of RNA expression of MET/RON pathway genes.** All 10 positive and negative regulators of MET/RON pathway are compared at the RNA expression level between cetuximab-sensitive CC cells and their cetuximab-resistant counterpart SC cells grown in 3D type-1 collagen. Analysis is performed on dataset published previously (10).

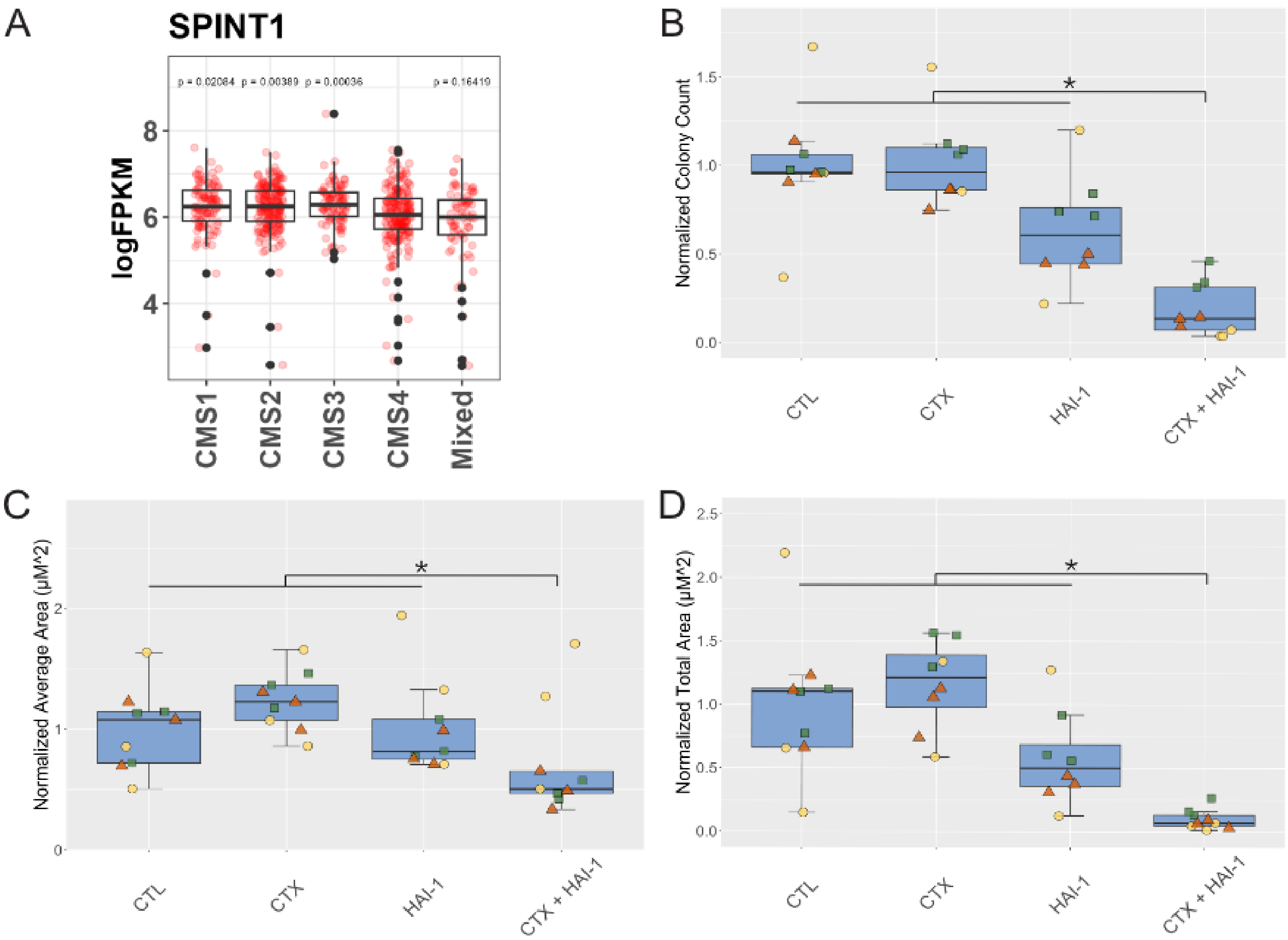


**Fig. S10. Quantification of CC-HGF 3D collagen cultures under treatment with cetuximab and recombinant soluble HAI-1. (A)** SPINT1 expression in TCGA CRC datasets (CMS classification). **(B,C,D)** Two thousand CC-HGF cells were seeded in 3D in type-I collagen and incubated with cetuximab (CTX, 3 µg/ml) alone or in combination with HAI-1 (10 µg/ml) as indicated for 14 days. Three independent experiments containing three replicates each are plotted for (B) normalized colony count, (C) normalized average colony area, and (D) normalized total colony area. Values are plotted as mean ± SEM; * indicates statistically significant differences (one-way ANOVA with Tukey's HSD post hoc test, $p < 0.05$).

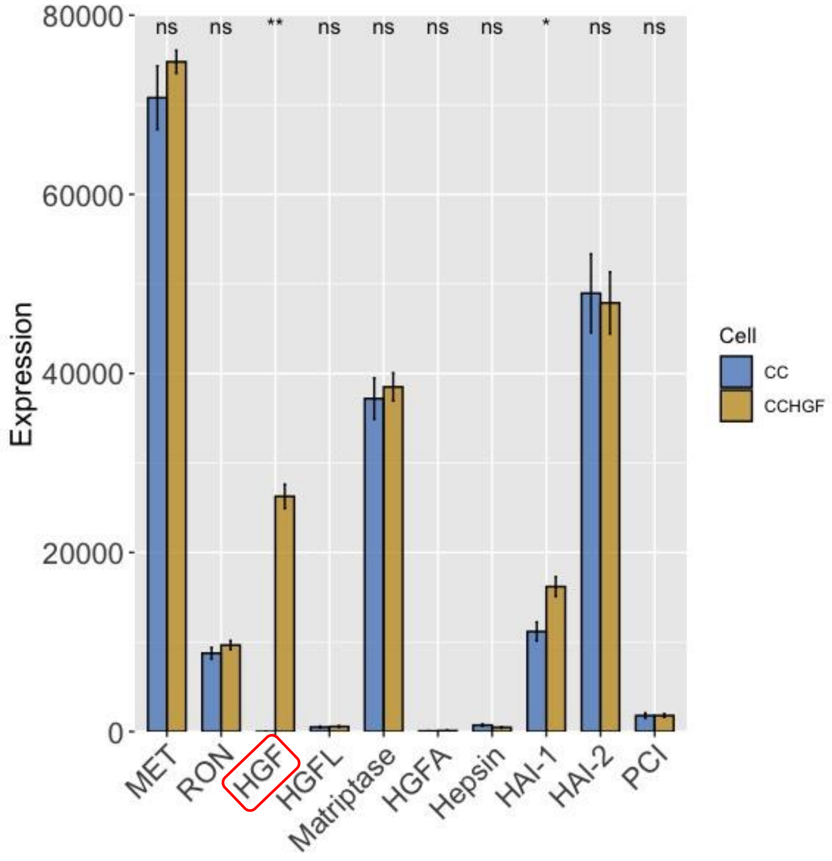


**Fig. S11. CC versus CC-HGF comparison of RNA expression of MET/RON pathway genes.** All 10 positive and negative regulators of MET/RON pathway are compared at the RNA expression level (RNAseq) between cetuximab-sensitive CC cells and their cetuximab-resistant counterpart CC-HGF cells grown in 3D type-1 collagen. Statistical analysis: ns = not significant; * = $p<0.05$; ** = $p<0.01$ (one-way ANOVA with Tukey's HSD post hoc test).

**Supplementary Movie 1. Crizotinib overcomes HGF-induced cetuximab resistance and loss of polarity.** Live-cell imaging of CC (top panel) and CC-HGF (bottom panel) colonies grown in 3D in type-1 collagen treated with cetuximab (CTX, 3 μg/ml) and/or crizotinib (CRIZ, 0.25 μM) as indicated on top. CC and CC-HGF colonies were imaged for 12 days with the ongoing treatments indicated. Quantification of 3D growth and morphology from several CC and CC-HGF colonies is included in Fig. 4A and 4D, respectively. Scale bars: 145 μm.

**Supplementary Table 1. RNA-seq comparison of RNA isolated from CC and CC-HGF 3D type-1 collagen cultures.** RNA was isolated at three separate times and expression of genes significantly; differentially expressed genes are shared in the table (absolute fold change >= 2 and FDR adjusted p value <= 0.05).
